# Supplementary material for: Thermally Stable Nitrothiacalixarene Chromophores: Conformational Study and Aggregation Behavior
Source: Int J Mol Sci. 2020 Sep 21;21(18):6916. doi: 10.3390/ijms21186916 (PMC7554919; doi:10.3390/ijms21186916)
Supplement: Supplementary file 1 [file ijms-21-06916-s001.pdf]

Thermally stable nitrothiacalixarene chromophores: conformational study and aggregation behavior

Anton Muravev[1, *], Tatiana Gerasimova[1], Robert Fayzullin[1], Olga Babaeva[1], Ildar Rizvanov[1], Ayrat Khamatgalimov[1], Marsil Kadirov[1], Sergey Katsyuba[1], Igor Litvinov[1] Shamil Latypov[1], Svetlana Solovieva[1, 2], and Igor Antipin[2]

[a]Arbuzov Institute of Organic and Physical Chemistry, FRC Kazan Scientific Center, Russian Academy of Sciences, Kazan, 420088 Russia

[b]Kazan Federal University, Kazan, 420008 Russia

Corresponding author e-mail: antonm@iopc.ru

Table of contents

Structures of the compounds	2
Compound **2a**	3
Compound **2b**	5
Compound **3**	7
Compound **5**	9
Compound **7**	11
Compound **7'**	13
Compound **8**	15
Compound **8'**	17
Compound **10**	18
Compound **12**	20
Computational data of relative energies of calixarene conformers	22
TG-DSC curves of the precursors	22
UV/Vis spectra of chromophores in solution and at air–water interface	25
Powder X-ray diffractometry data	26
Computational data of UV/Vis spectra of nitrothiacalixarene **3**	27

* Corresponding author. Tel.: +7-843-273-9365; fax: +7-843-273-1872; e-mail: antonm@iopc.ru

Structures of the compounds

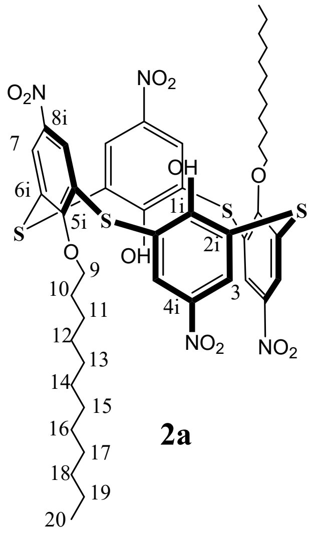

2a

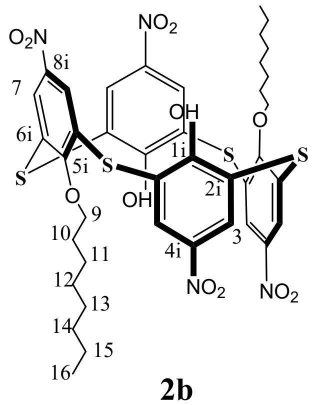

2b

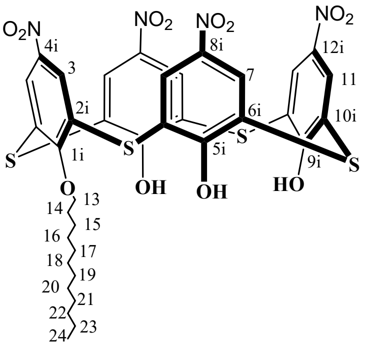

3

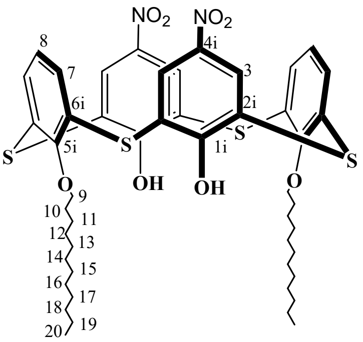

8

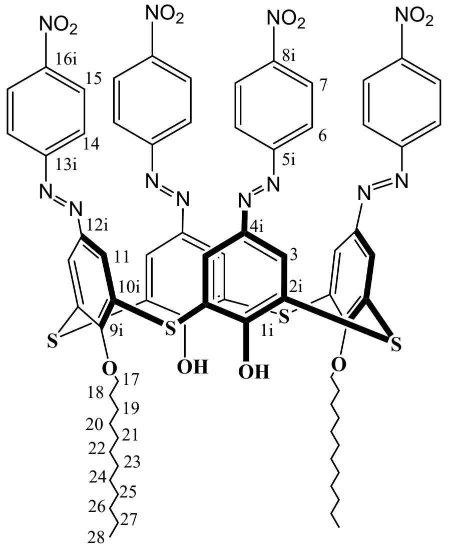

5

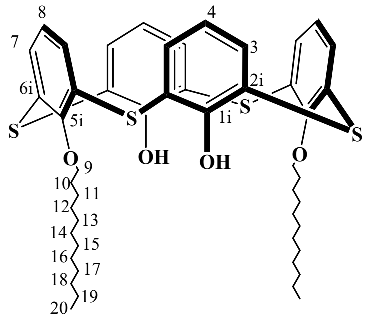

7

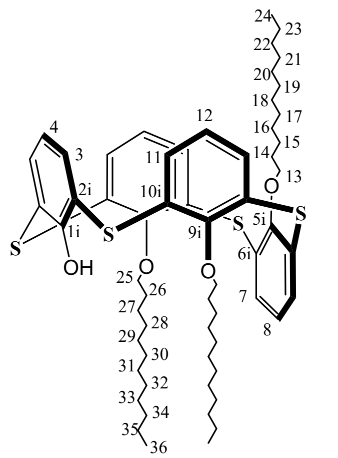

7'

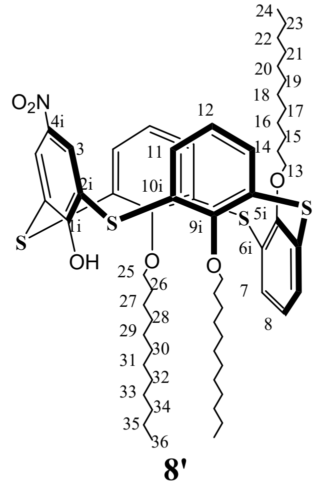

8'

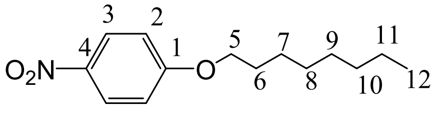

10

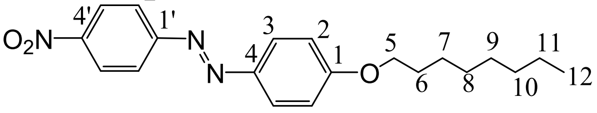

12

Compound 2a

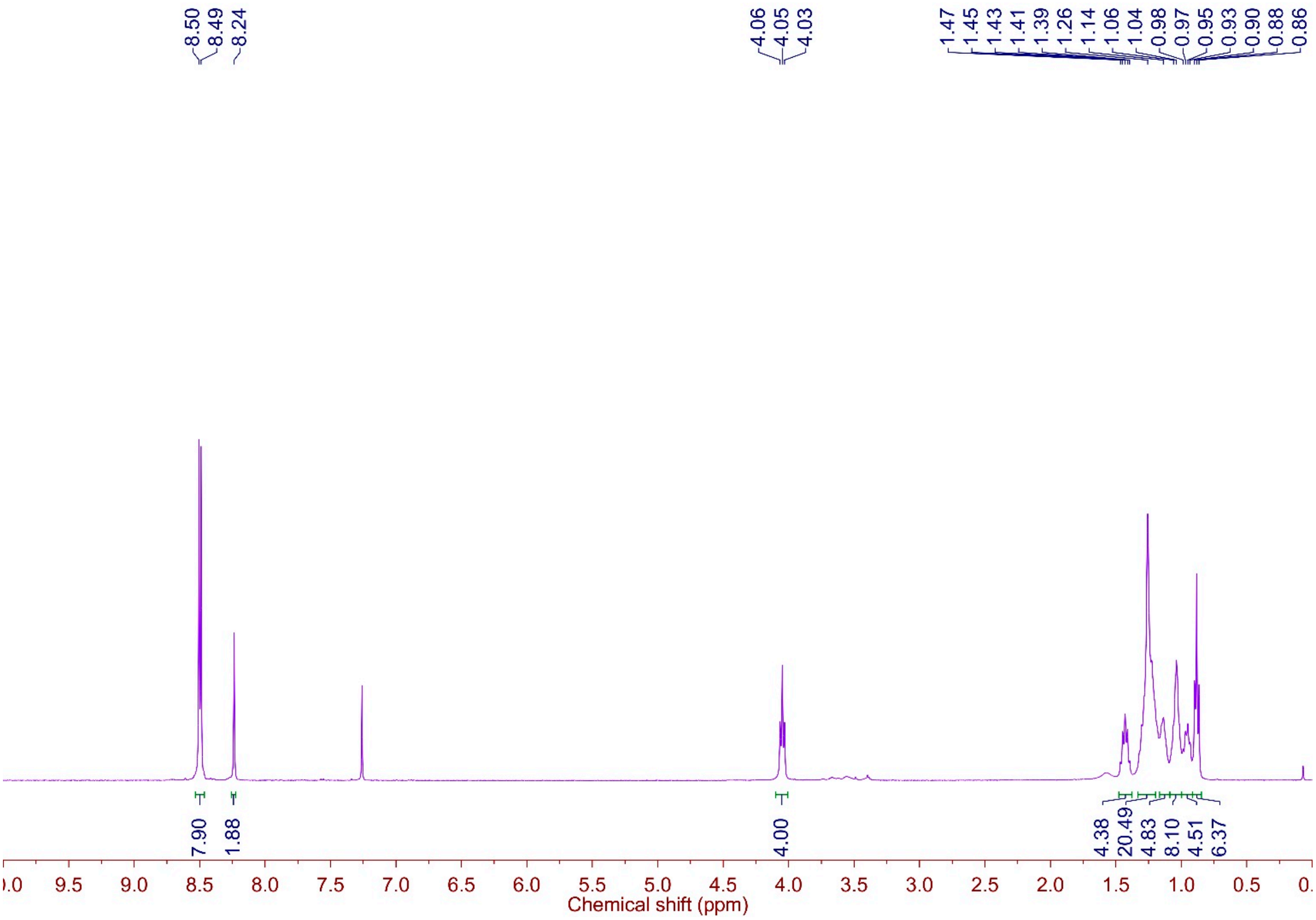

Fig. S1. ^{1}H NMR spectrum of compound **2a** ($CDCl_3$, 400 MHz, 298 K).

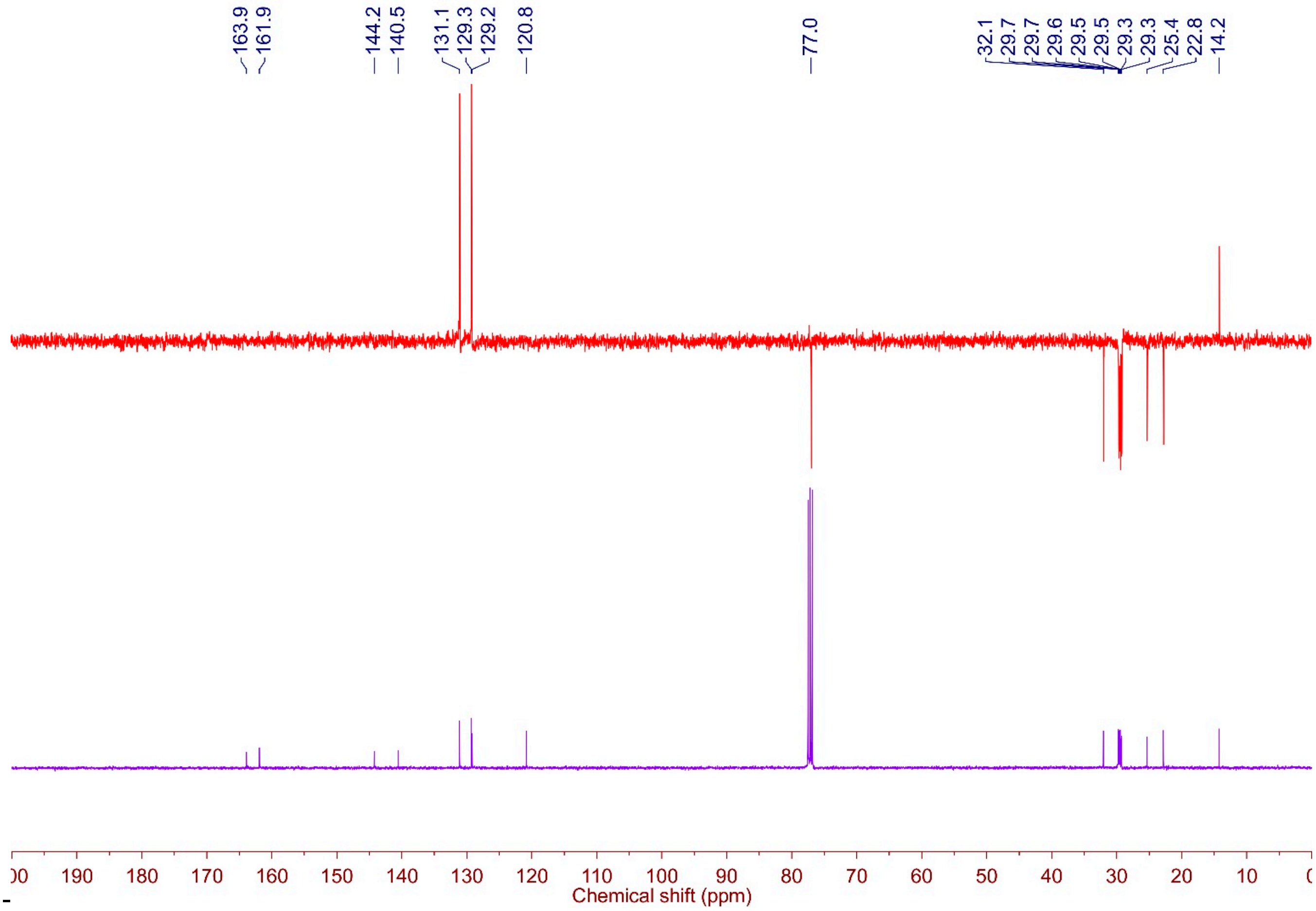

Fig. S2. ^{13}C NMR spectrum and DEPT-135 experiment of compound **2a** ($CDCl_3$, 100 MHz, 298 K).

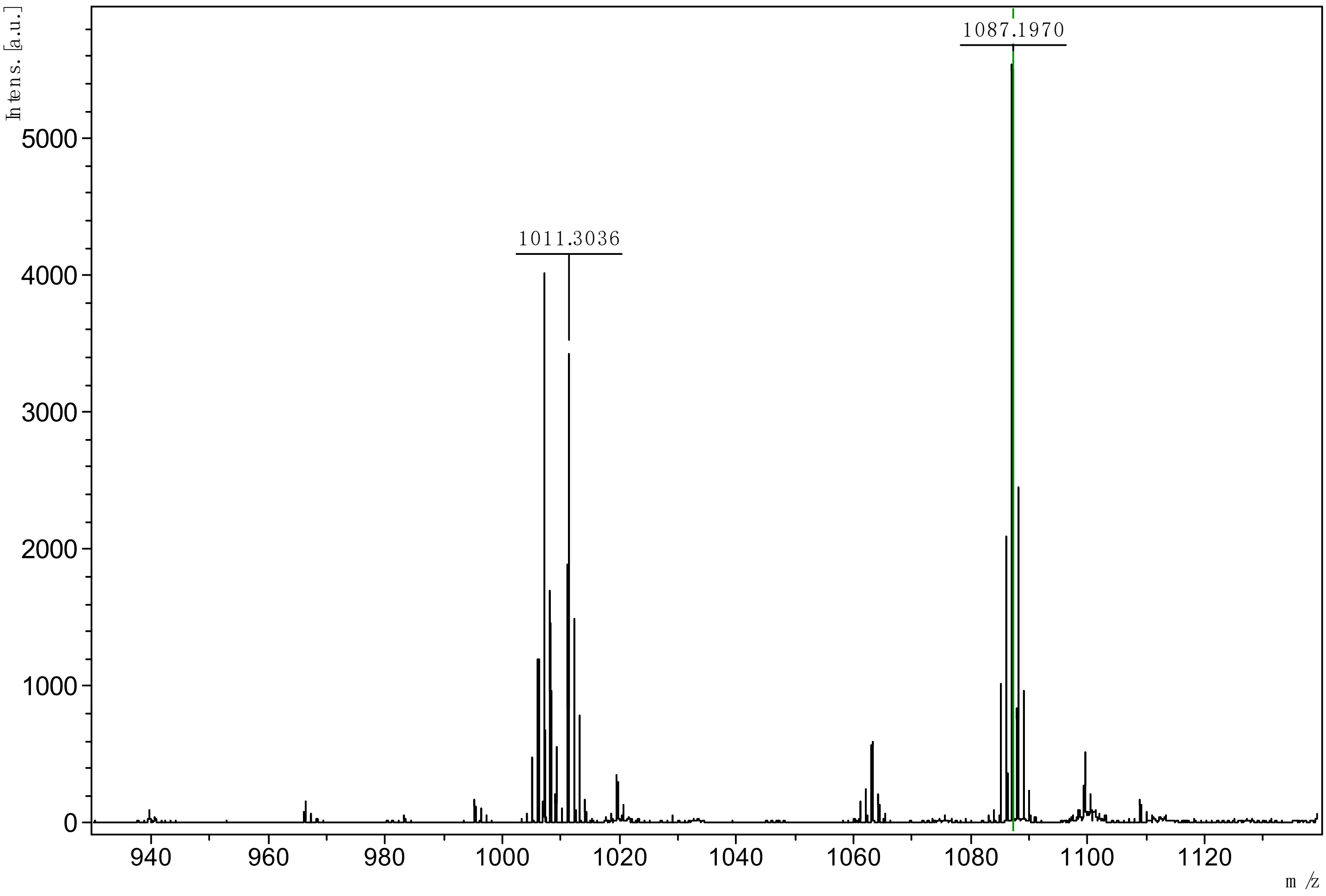

Fig. S3. HRMS spectrum of compound **2a** (*p*-nitroaniline, standard).

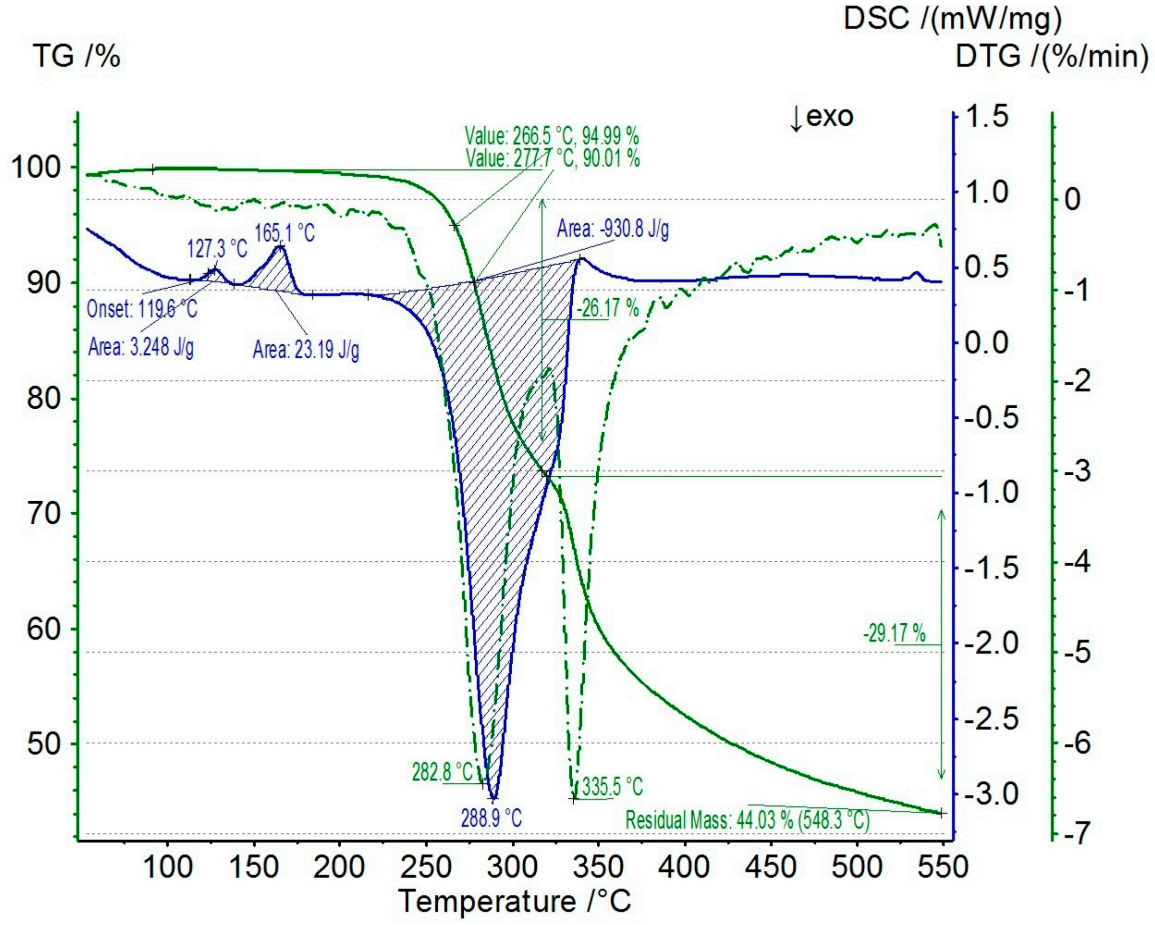

Fig. S4. TG/DTG-DSC/dDSC curves of compound **2a**.

Compound 2b

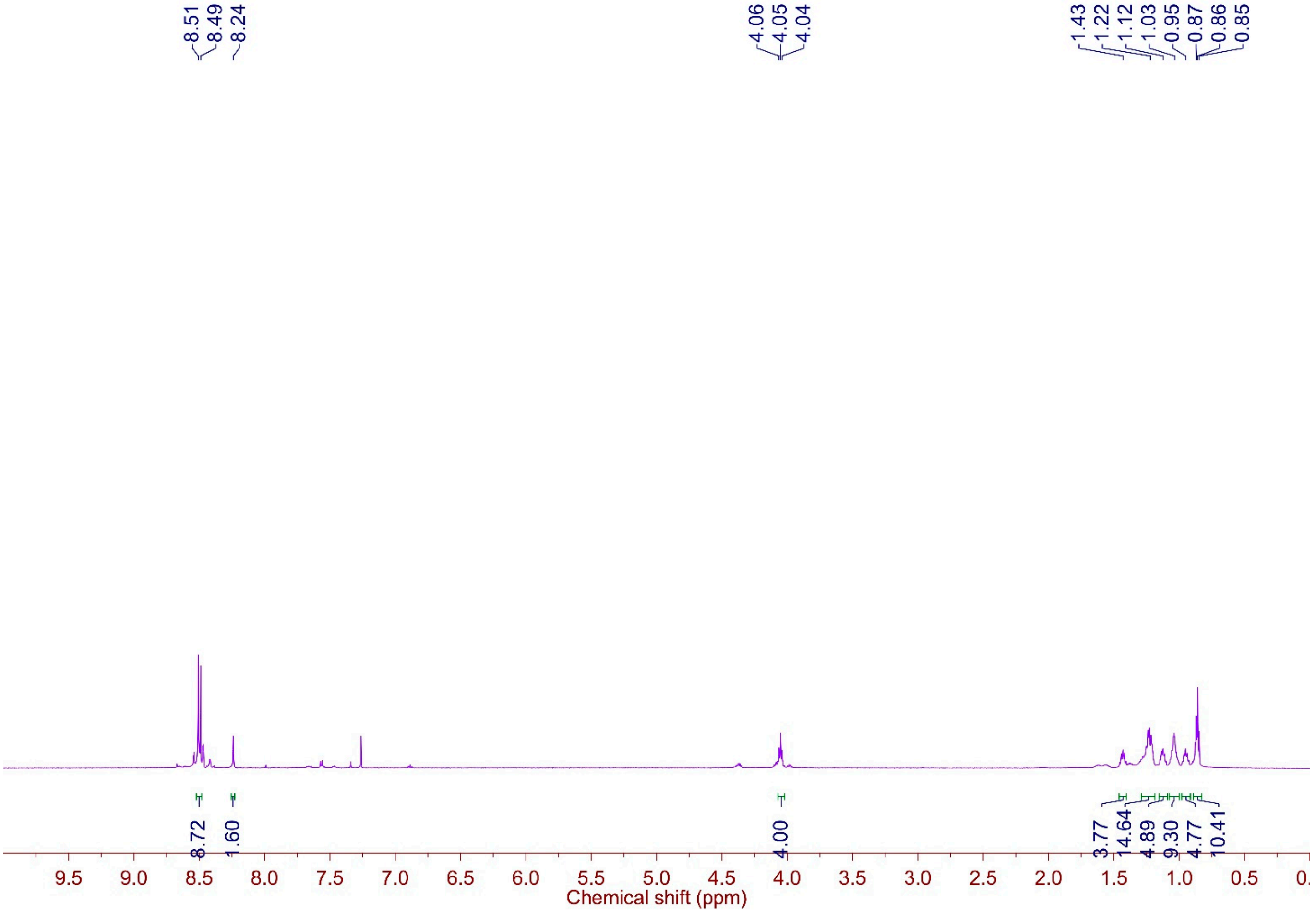

Fig. S5. ^{1}H NMR spectrum of compound **2b** ($CDCl_3$, 600 MHz, 298 K).

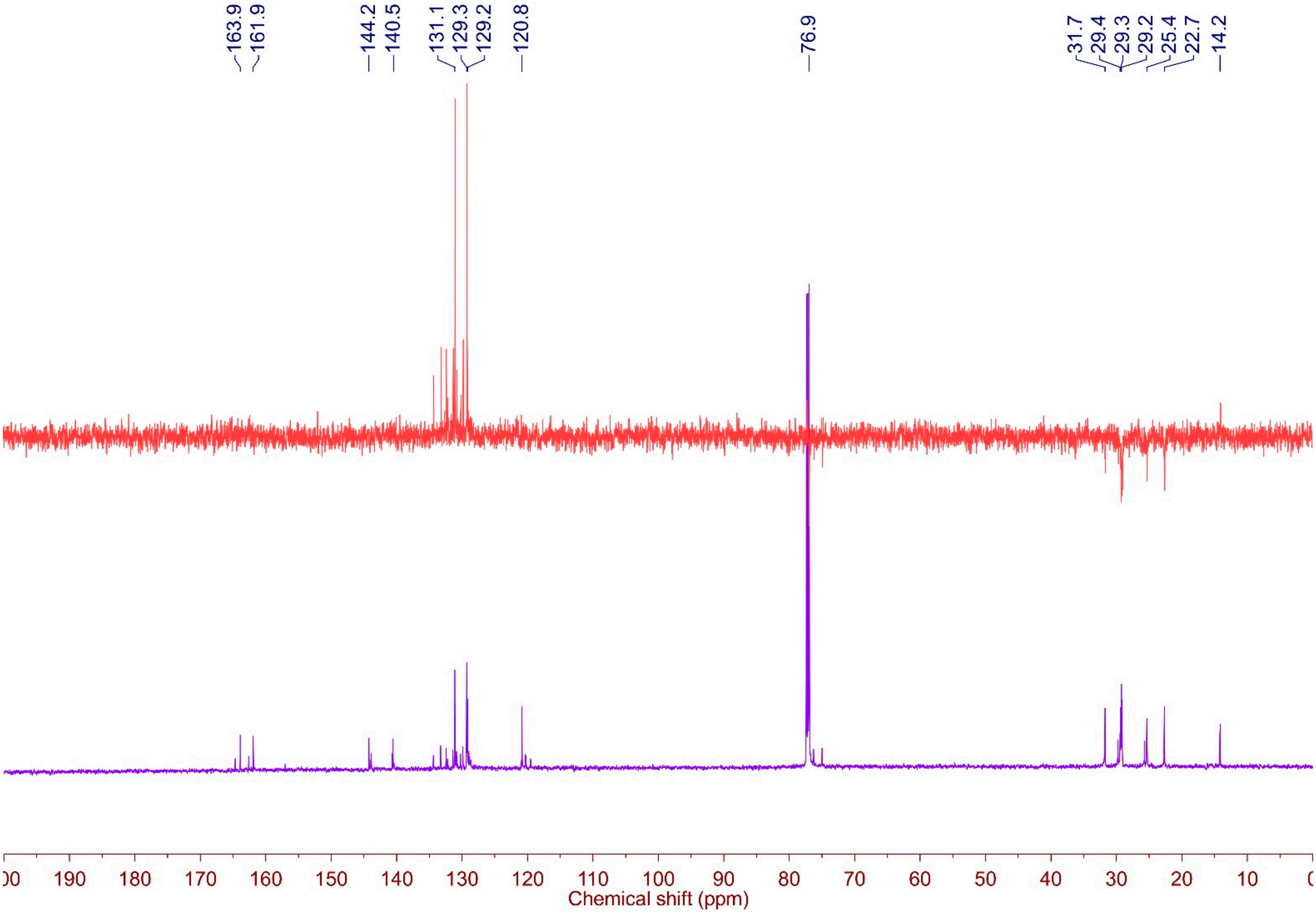

Fig. S6. ^{13}C NMR spectrum and DEPT-135 experiment of compound **2b** ($CDCl_3$, 150 MHz, 298 K).

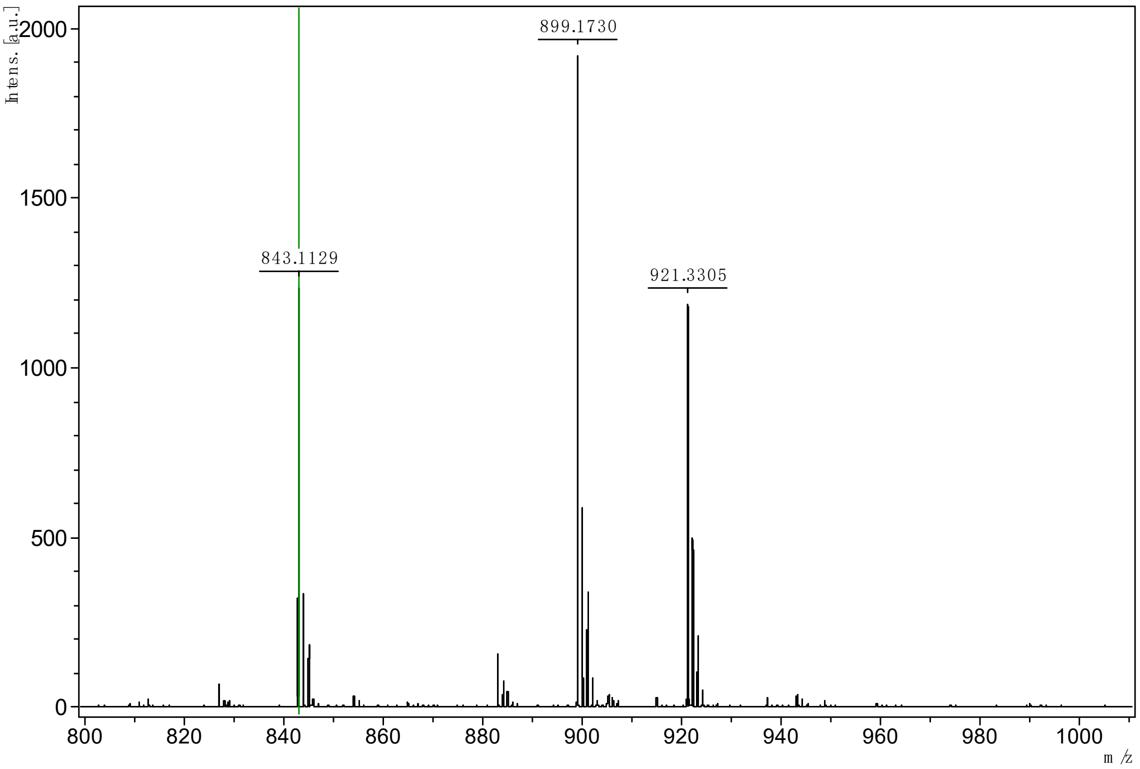

Fig. S7. HRMS spectrum of compound **2b** (*p*-nitroaniline, standard).

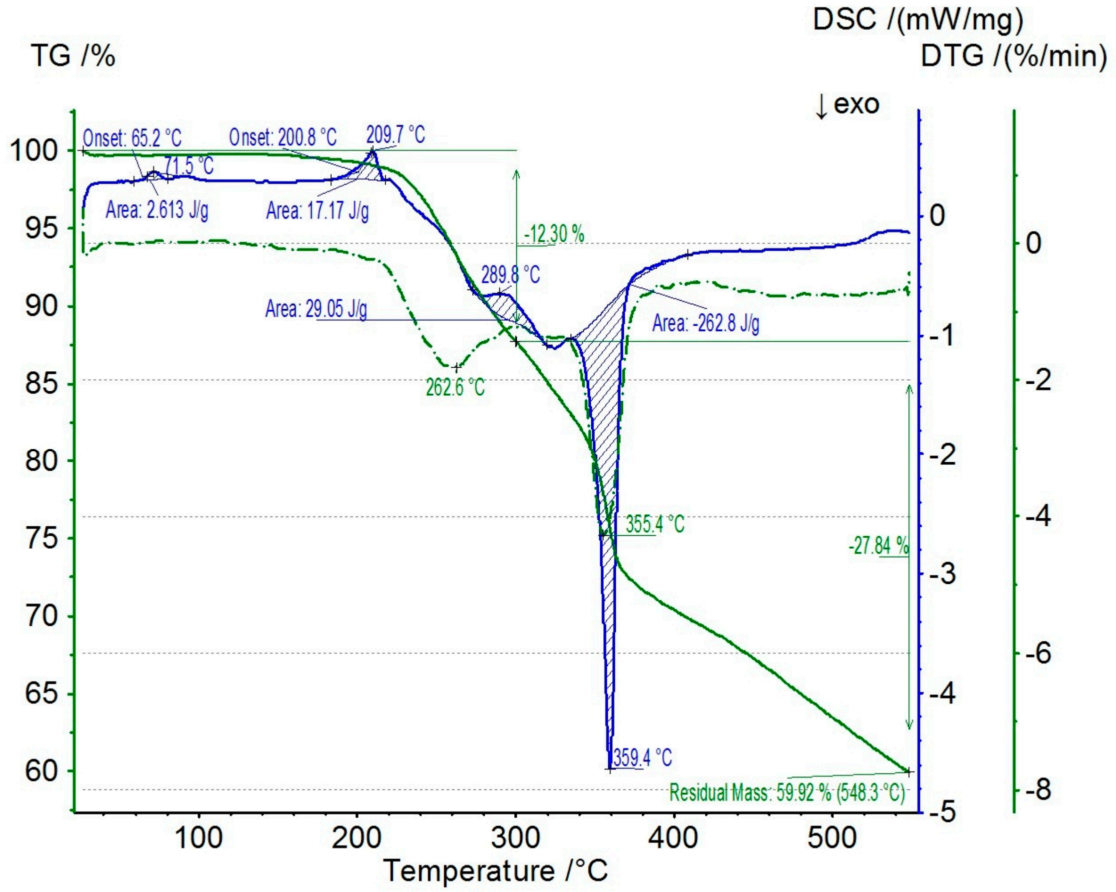

Fig. S8. TG/DTG-DSC curves of compound **2b**.

Compound 3

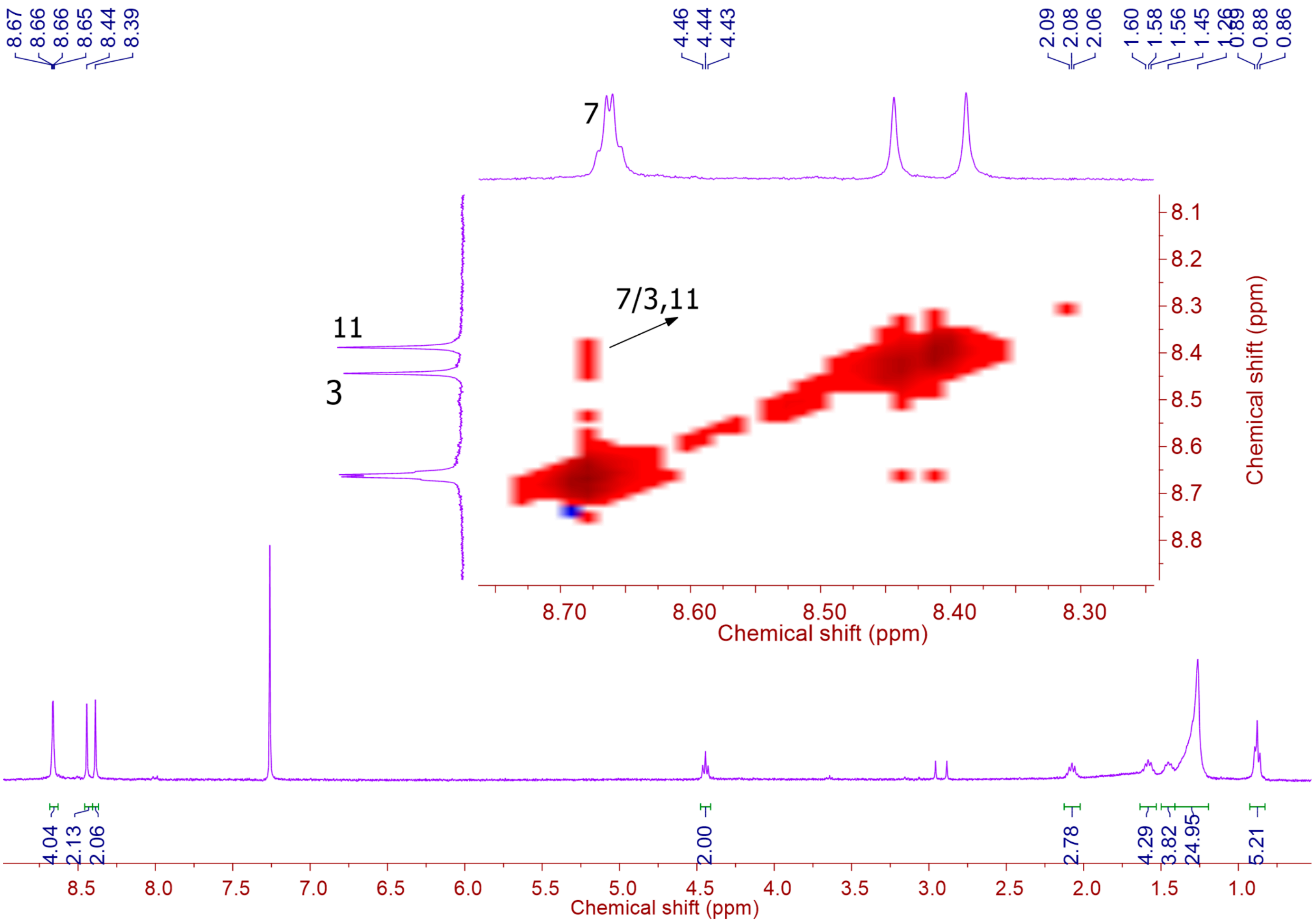

Fig. S9. ^{1}H NMR spectrum of compound **3** ($CDCl_3$, 400 MHz, 298 K) and its 2D NOESY experiment (8.8–8.2 ppm).

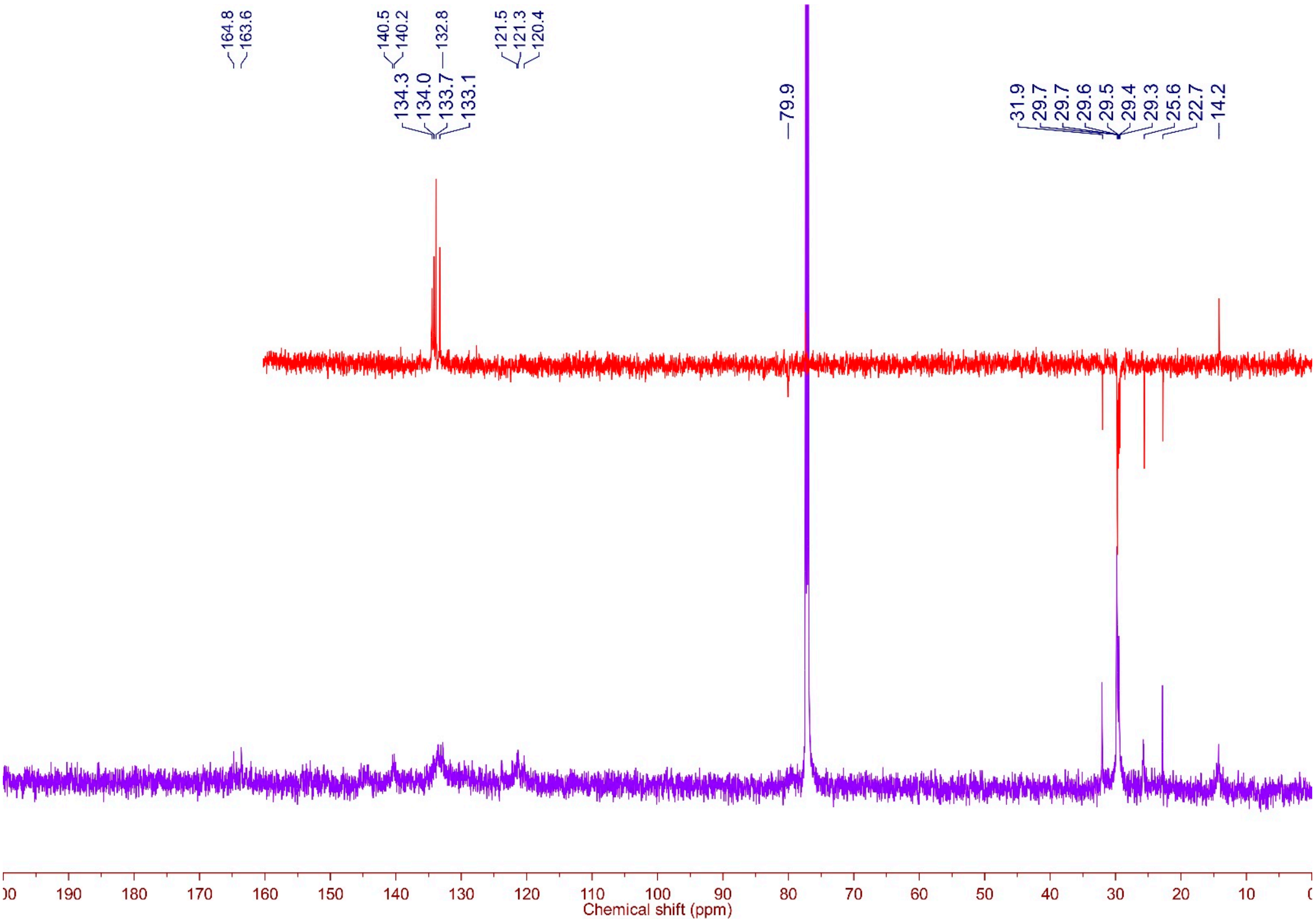

Fig. S10. ^{13}C NMR spectrum and DEPT-135 experiment of compound **3** ($CDCl_3$, 100 MHz, 298 K).

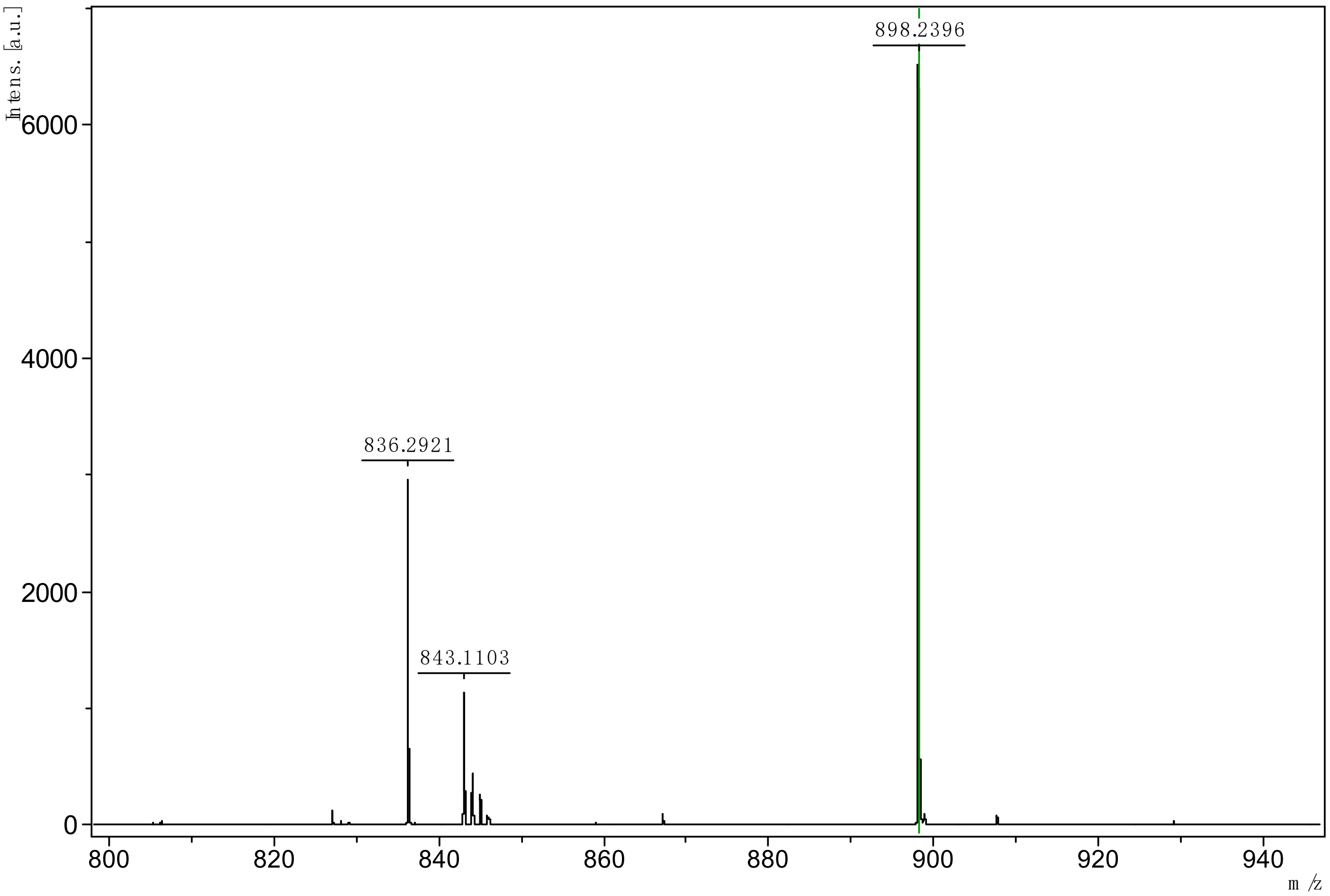

Fig. S11. HRMS spectrum of compound **3** (*p*-nitroaniline, standard).

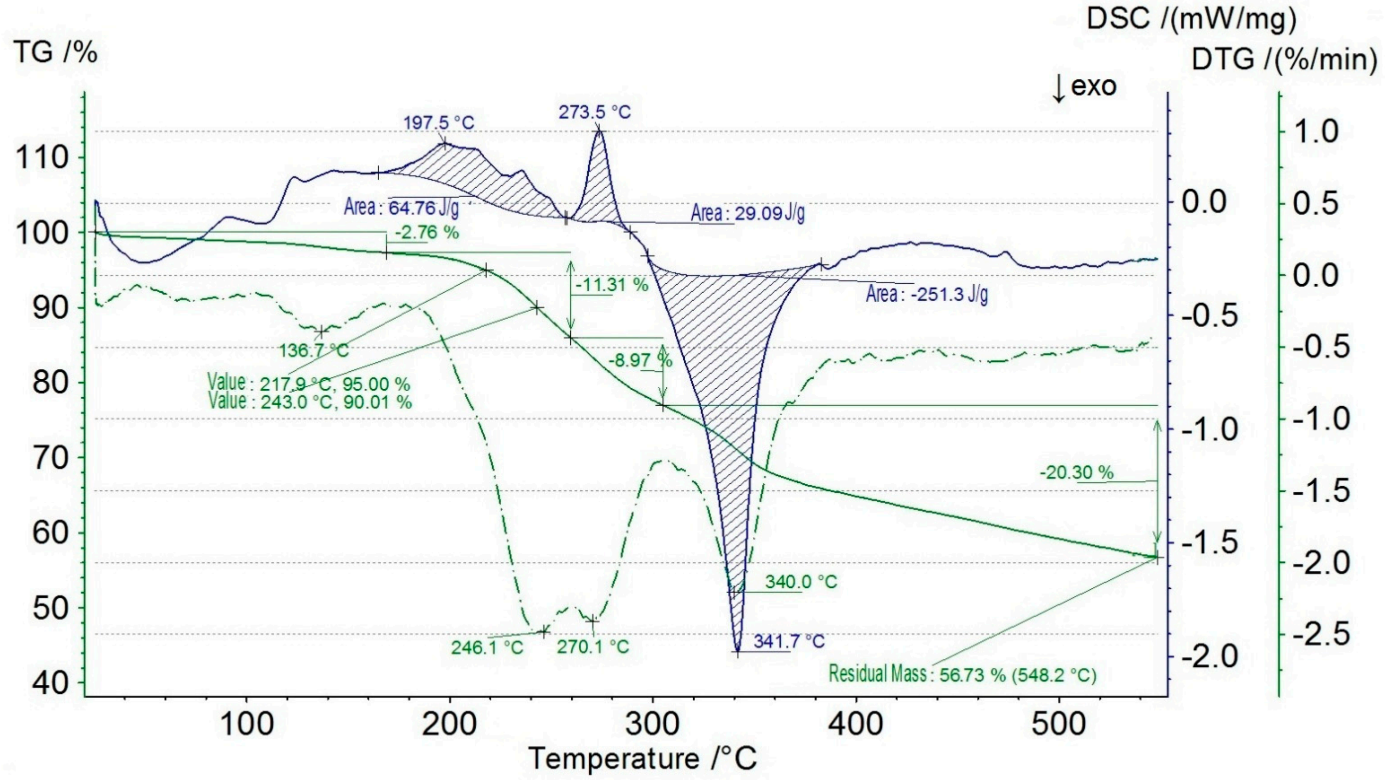

Fig. S12. TG/DTG-DSC curves of compound **3**.

Compound 5

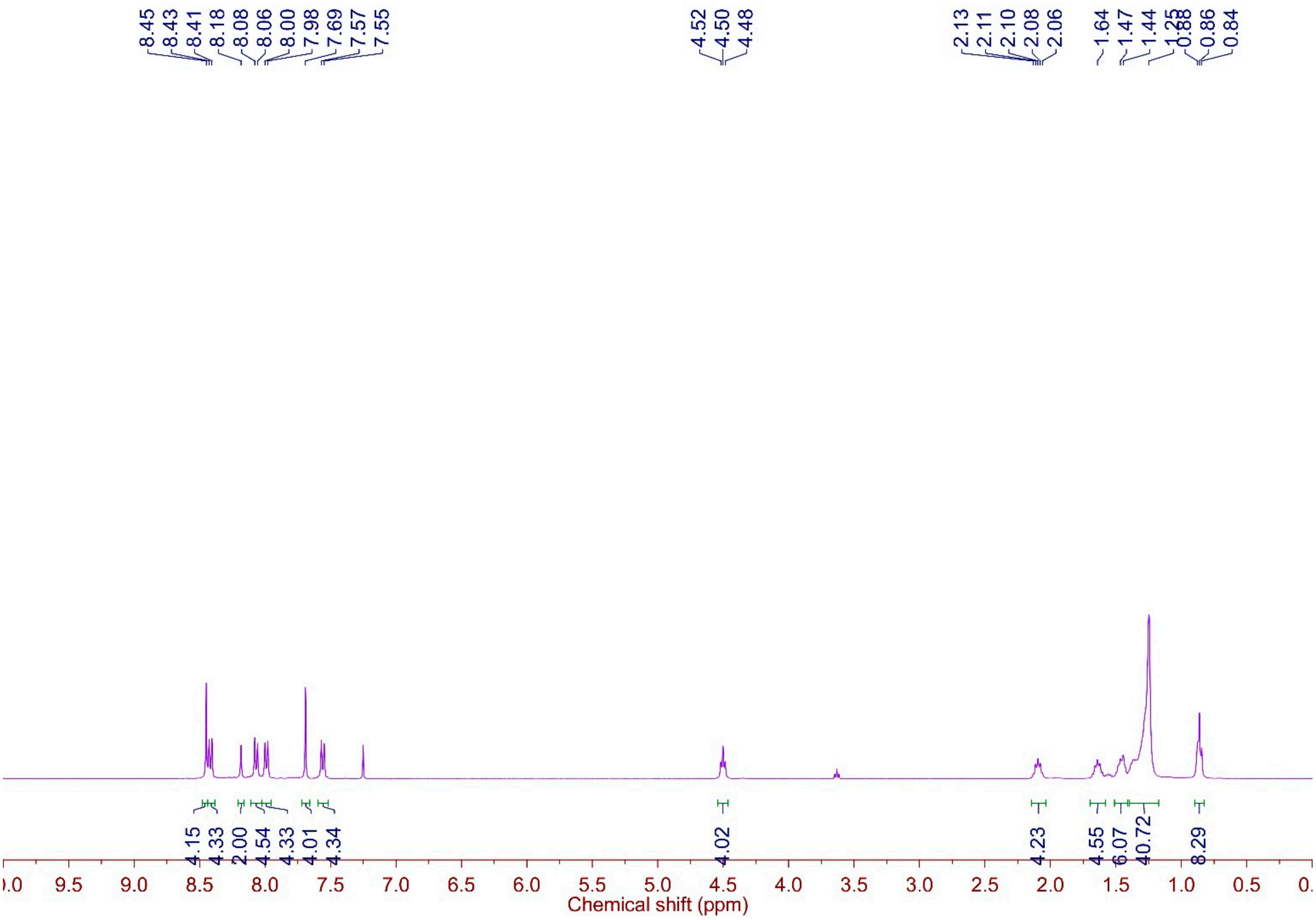

Fig. S13. ^{1}H NMR spectrum of compound **5** ($CDCl_3$, 400 MHz, 298 K).

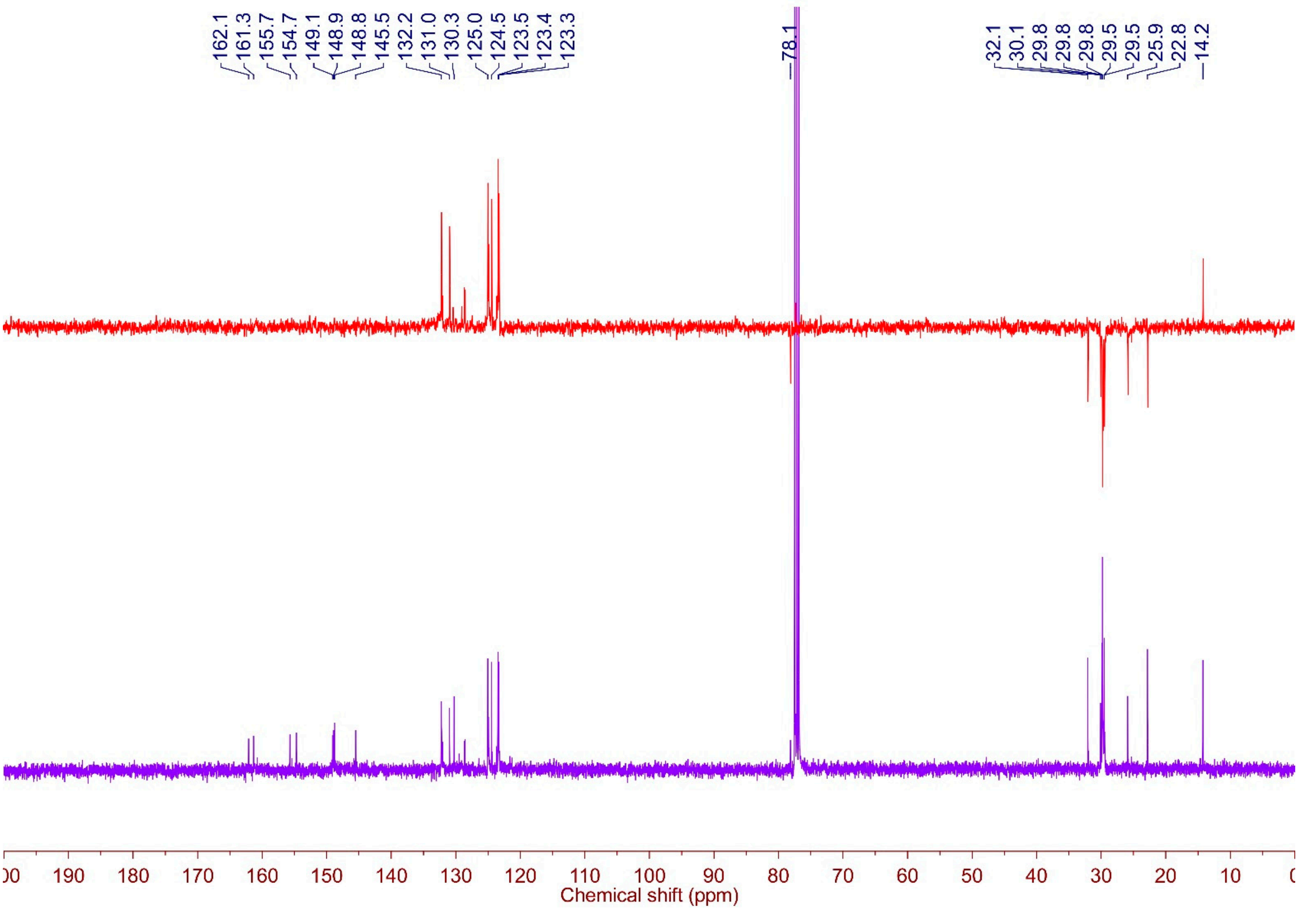

Fig. S14. ^{13}C NMR spectrum and DEPT-135 experiment of compound **5** ($CDCl_3$, 100 MHz, 298 K).

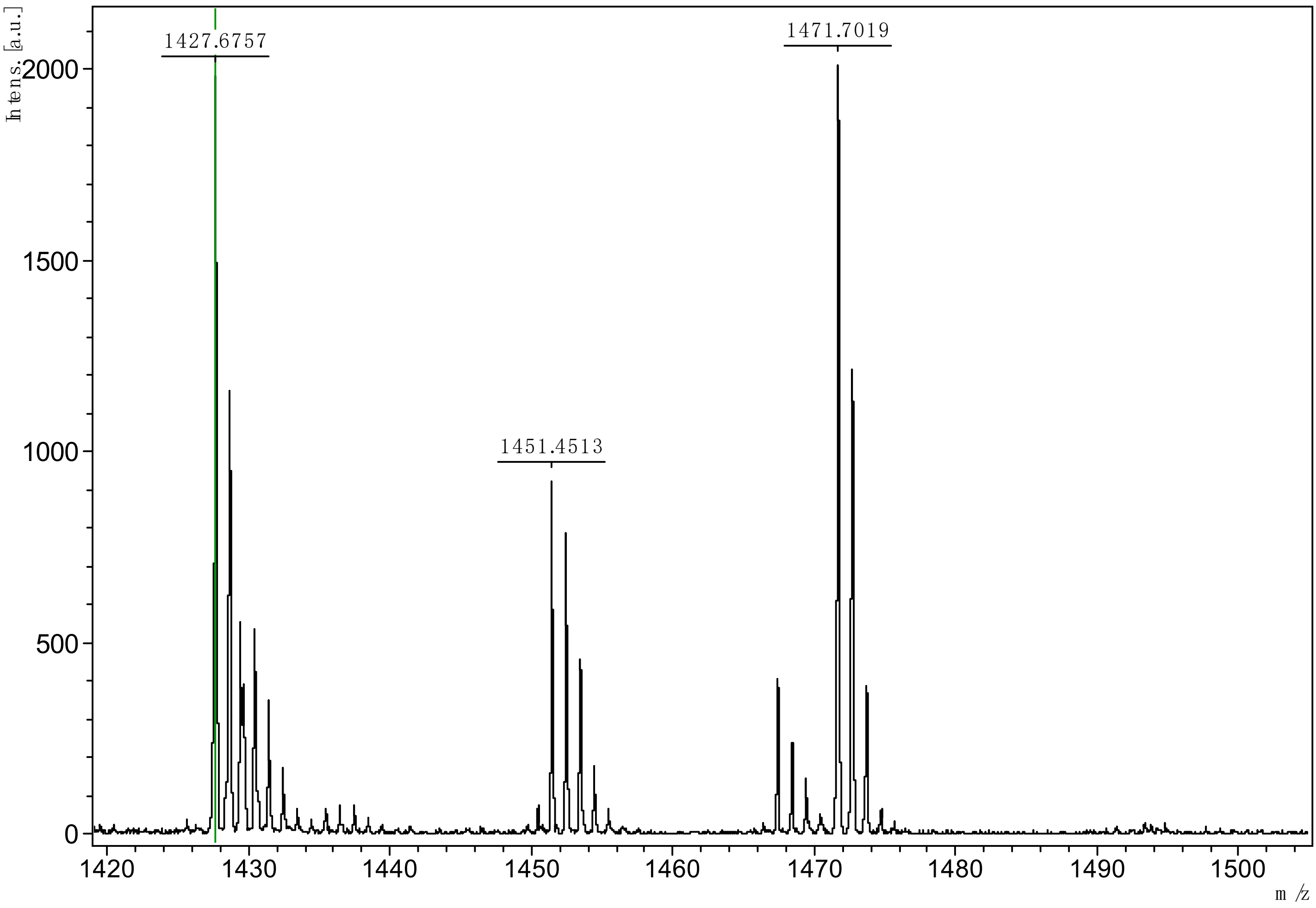

Fig. S15. HRMS spectrum of compound **5** (*p*-nitroaniline, standard).

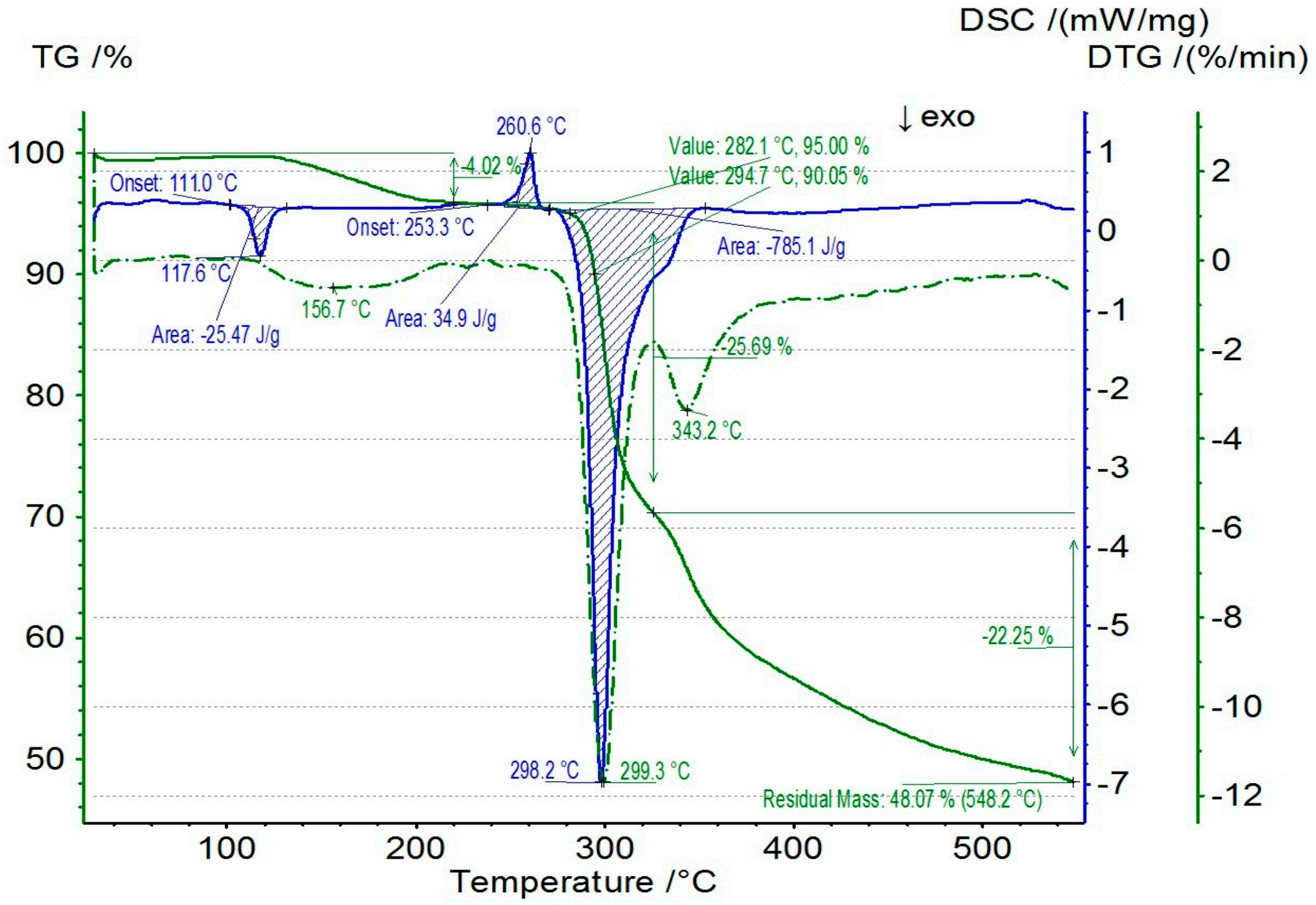

Fig. S16. TG/DTG-DSC curves of compound **5**.

Compound 7

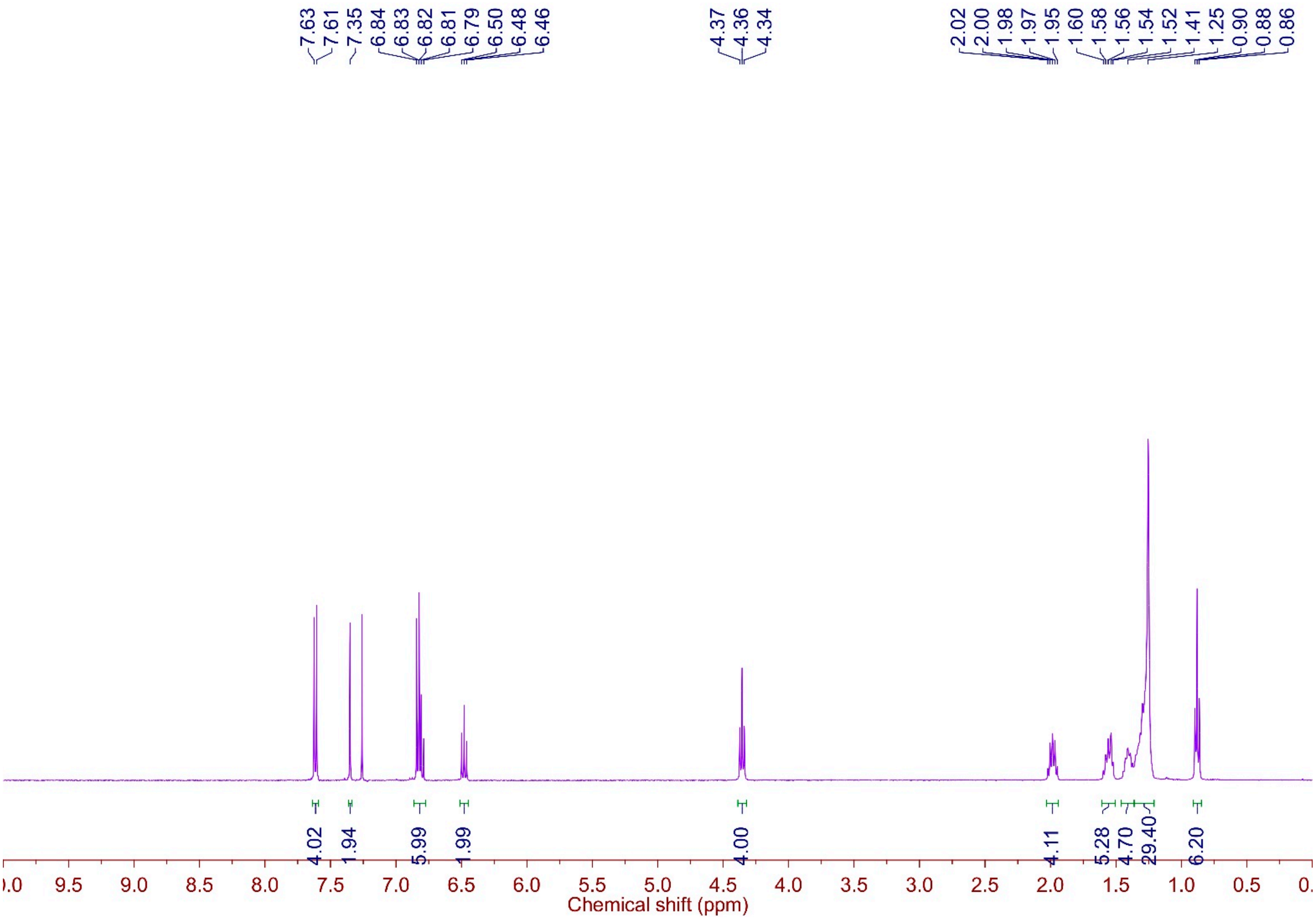

Fig. S17. ^{1}H NMR spectrum of compound **7** ($CDCl_3$, 400 MHz, 298 K).

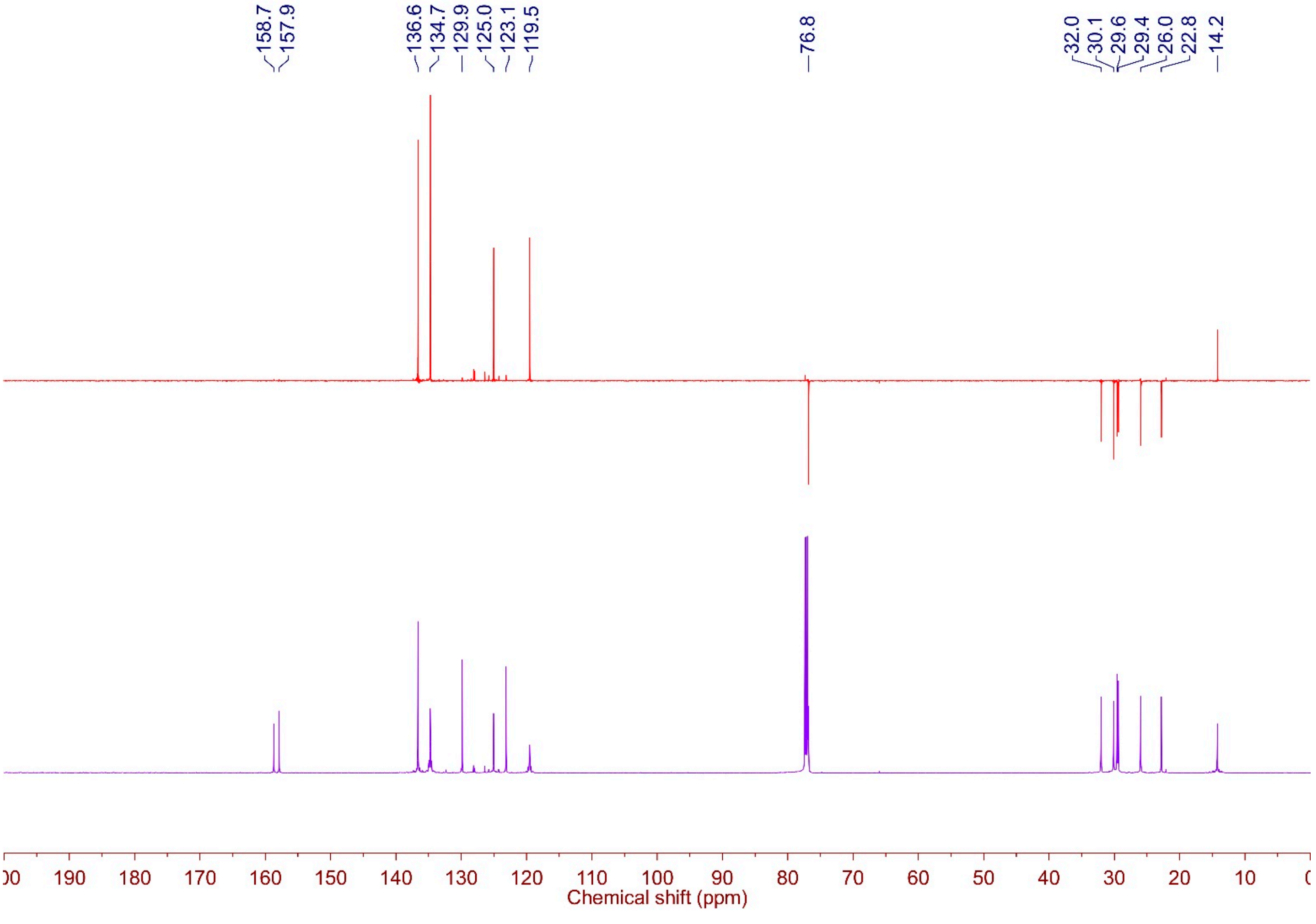

Fig. S18. ^{13}C NMR spectrum and DEPT-135 experiment of compound **7** ($CDCl_3$, 100 MHz, 298 K).

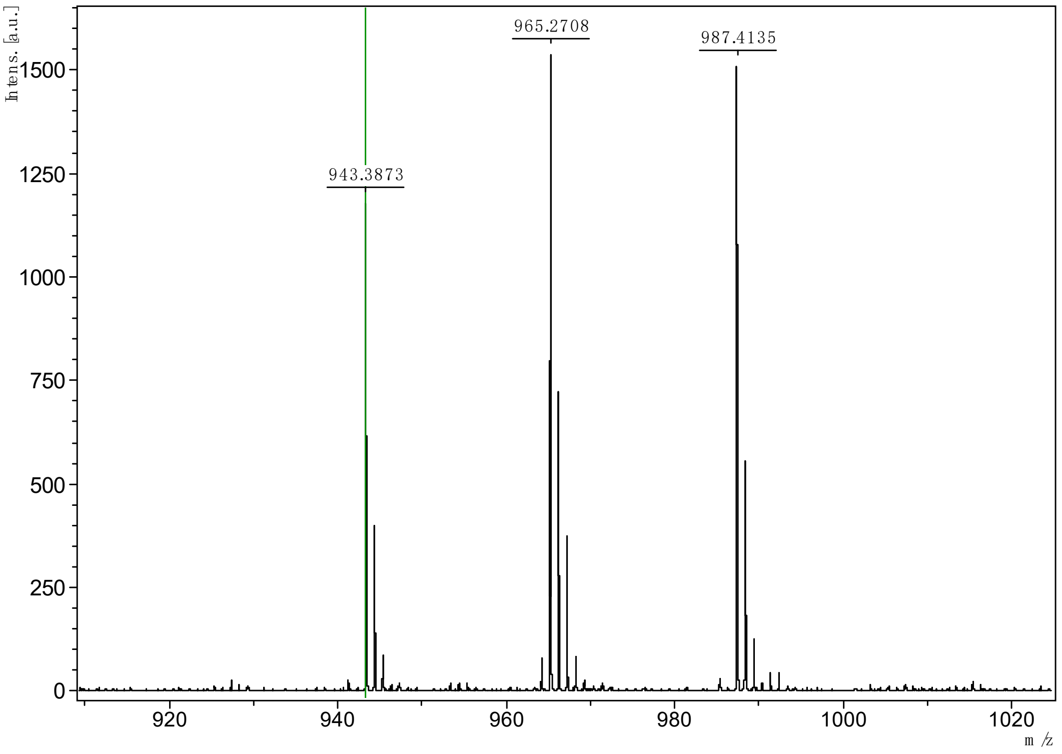

Fig. S19. HRMS spectrum of compound **7** (*p*-nitroaniline, standard).

Compound 7’

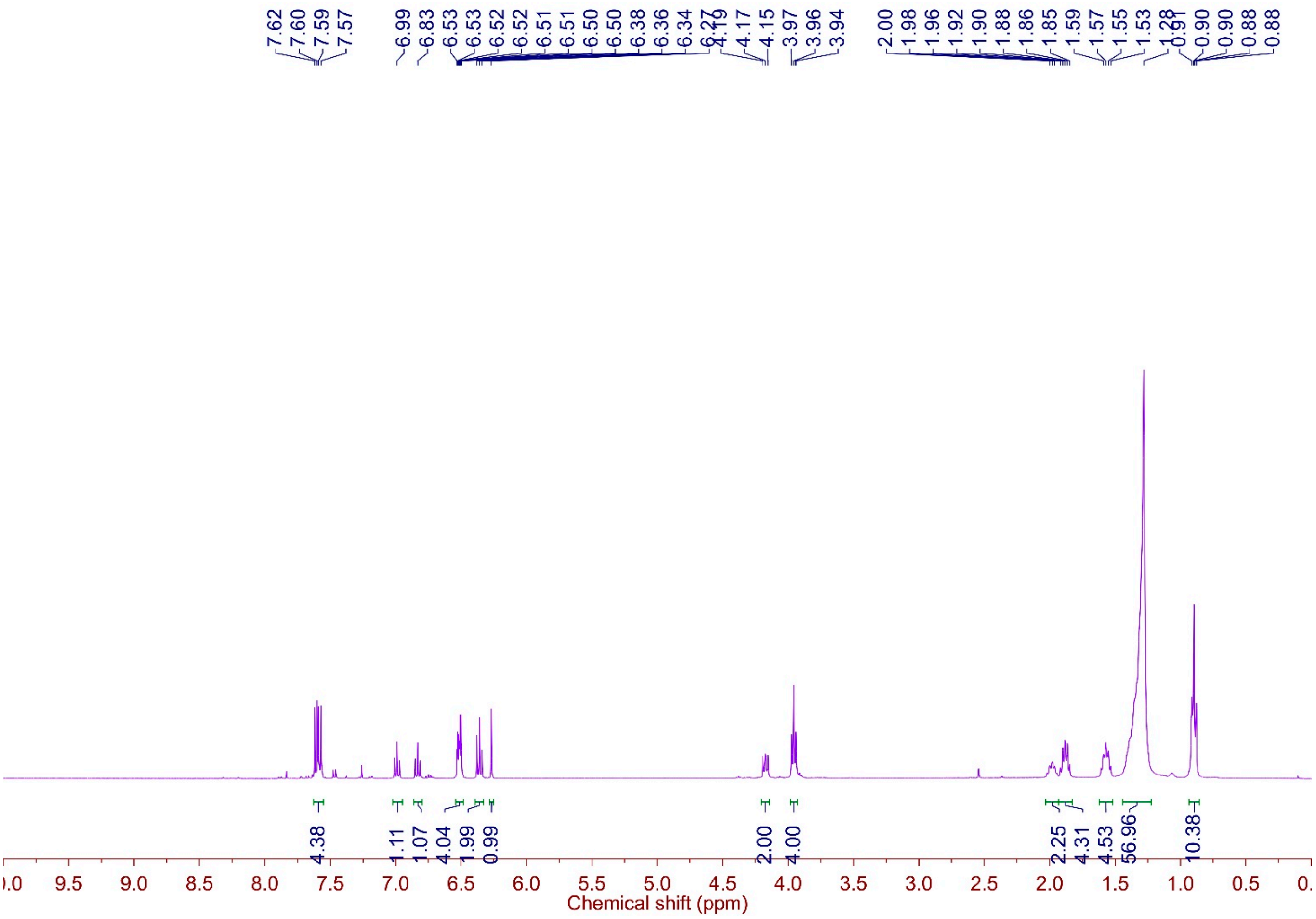

Fig. S20. ^{1}H NMR spectrum of compound **7’** ($CDCl_3$, 400 MHz, 298 K).

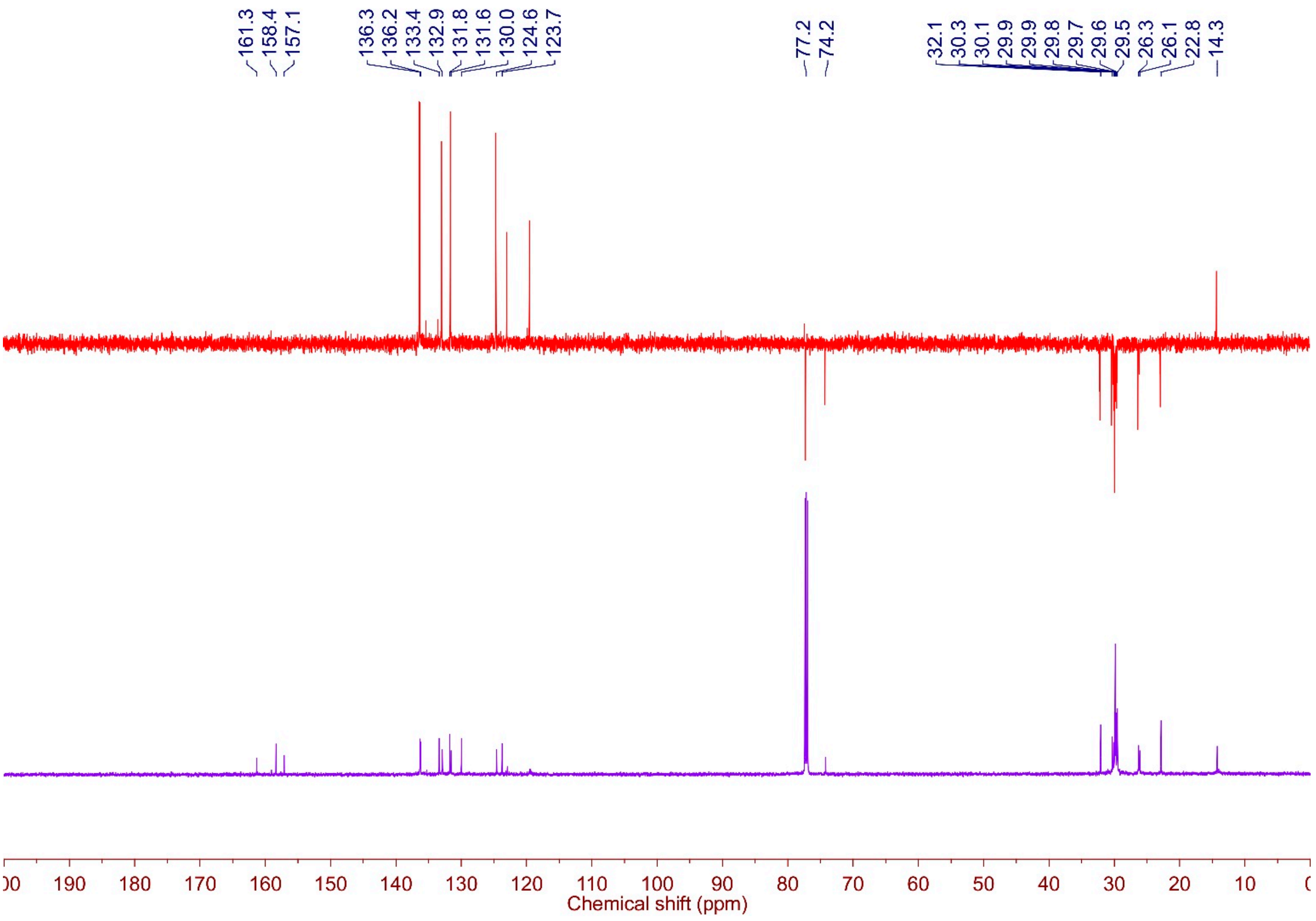

Fig. S21. ^{13}C NMR spectrum and DEPT-135 experiment of compound **7’** ($CDCl_3$, 100 MHz, 298 K).

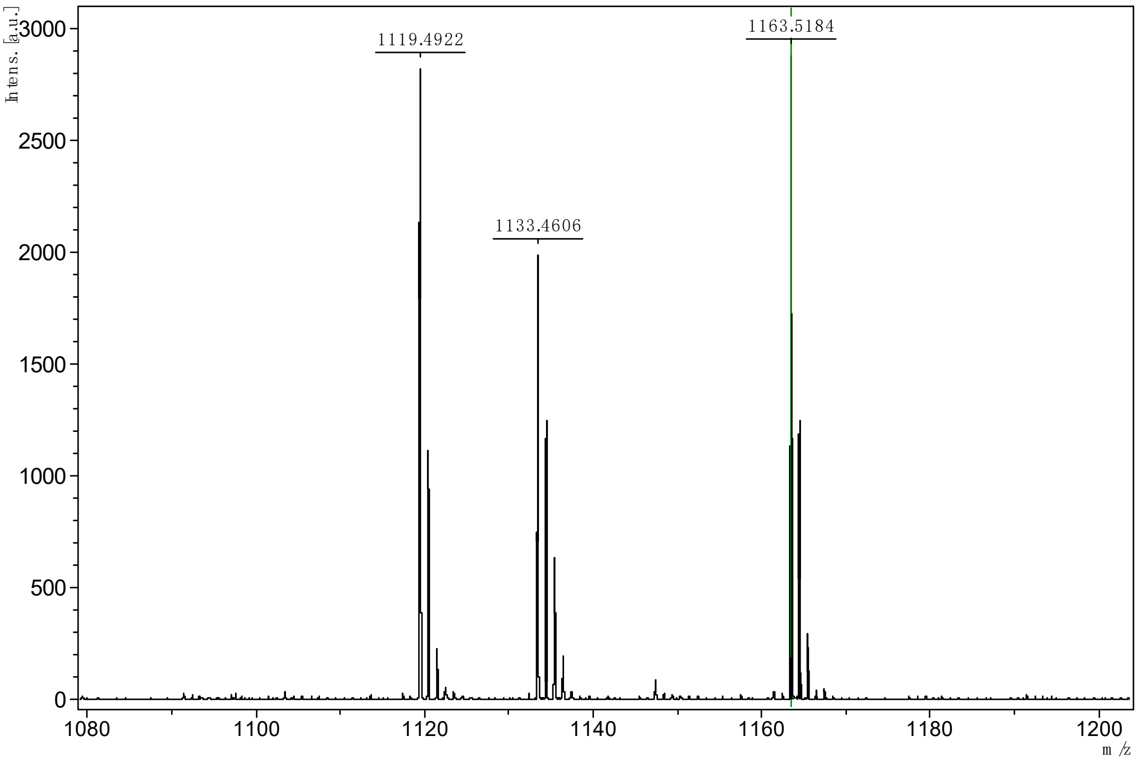

Fig. S22. HRMS spectrum of compound **7’** (*p*-nitroaniline, standard).

Compound 8

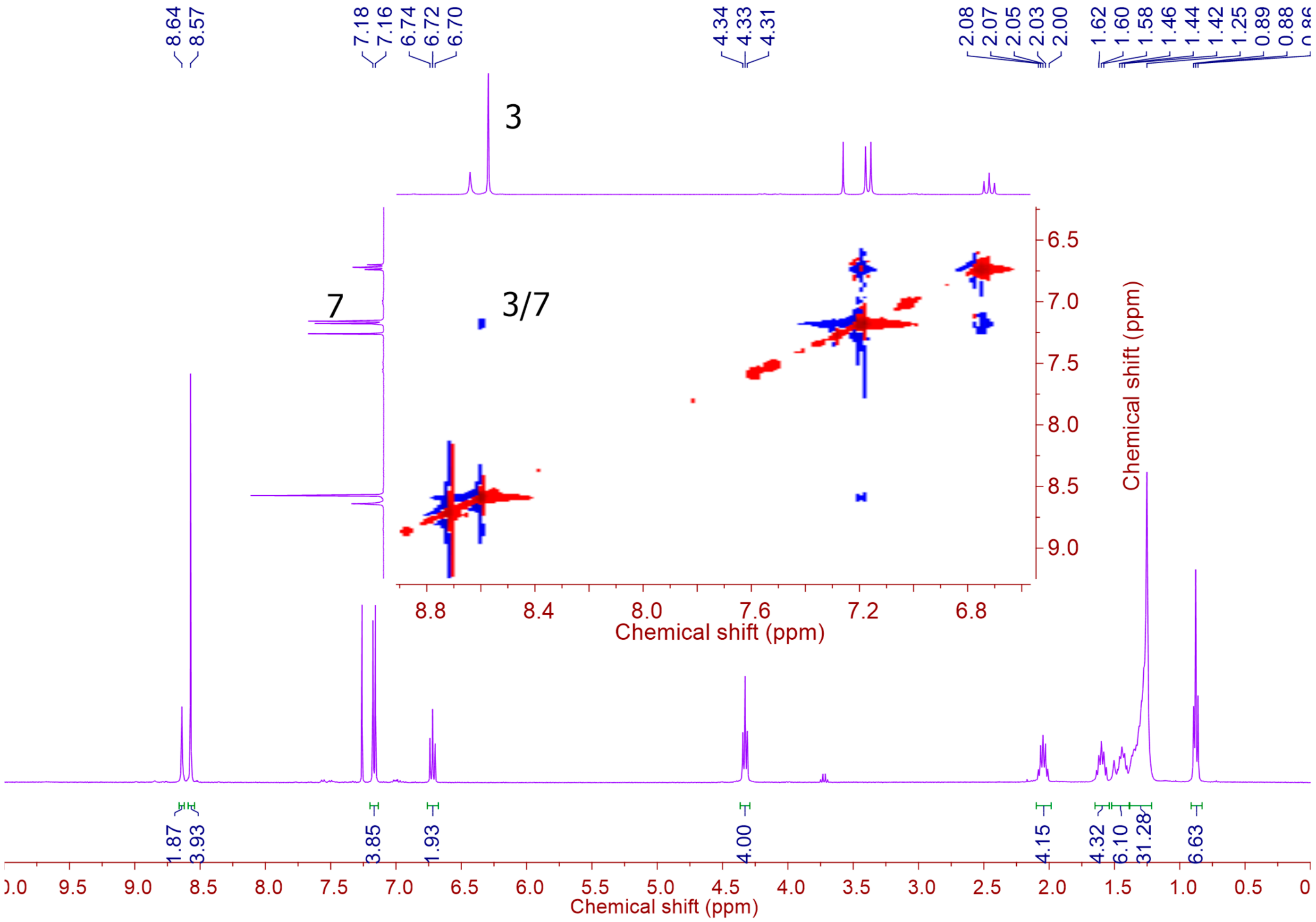

Fig. S23. ^{1}H NMR spectrum of compound **8** ($CDCl_3$, 400 MHz, 298 K) and its 2D NOESY experiment (8.9–6.7 ppm).

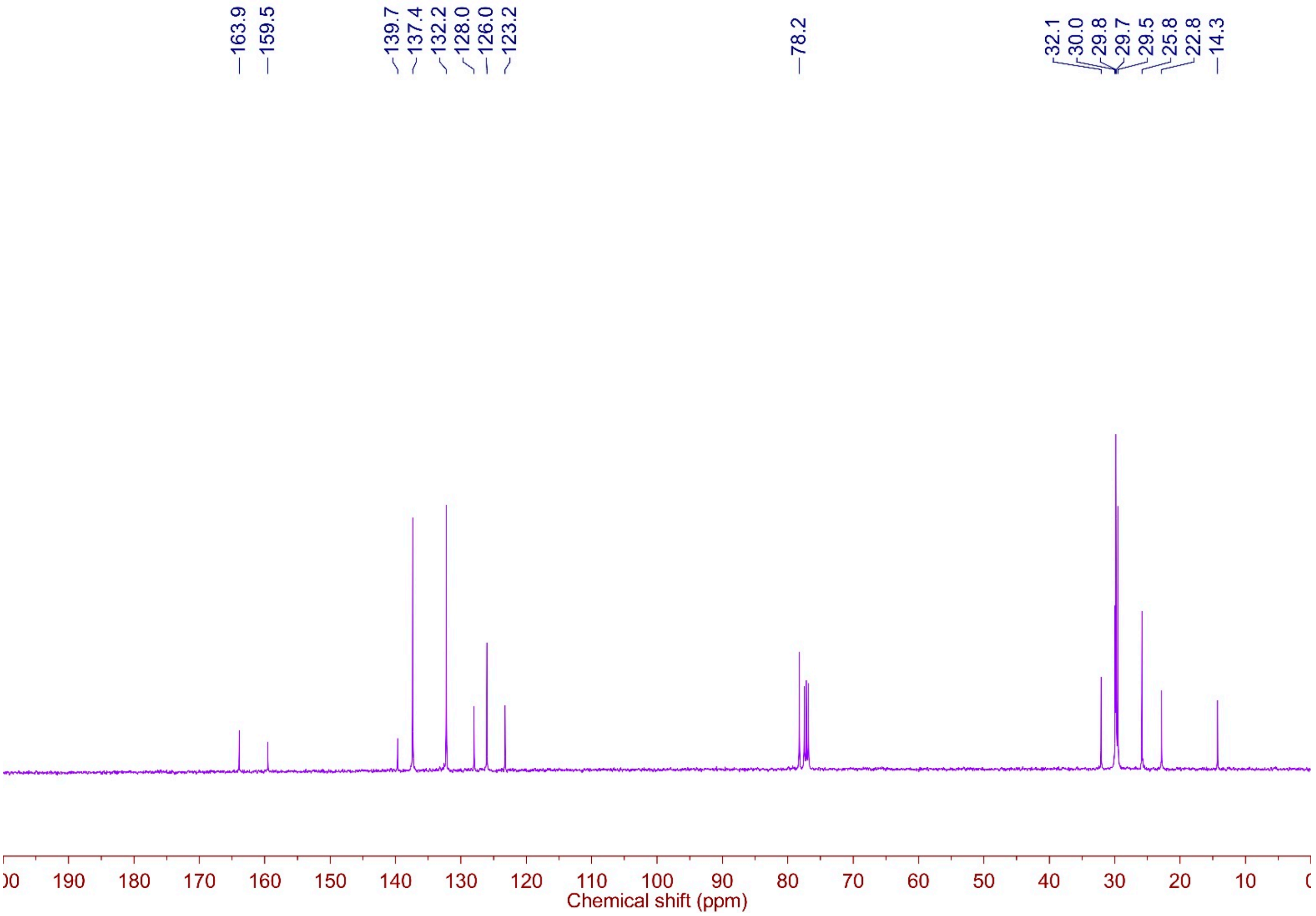

Fig. S24. ^{13}C NMR spectrum of compound **8** ($CDCl_3$, 100 MHz, 298 K).

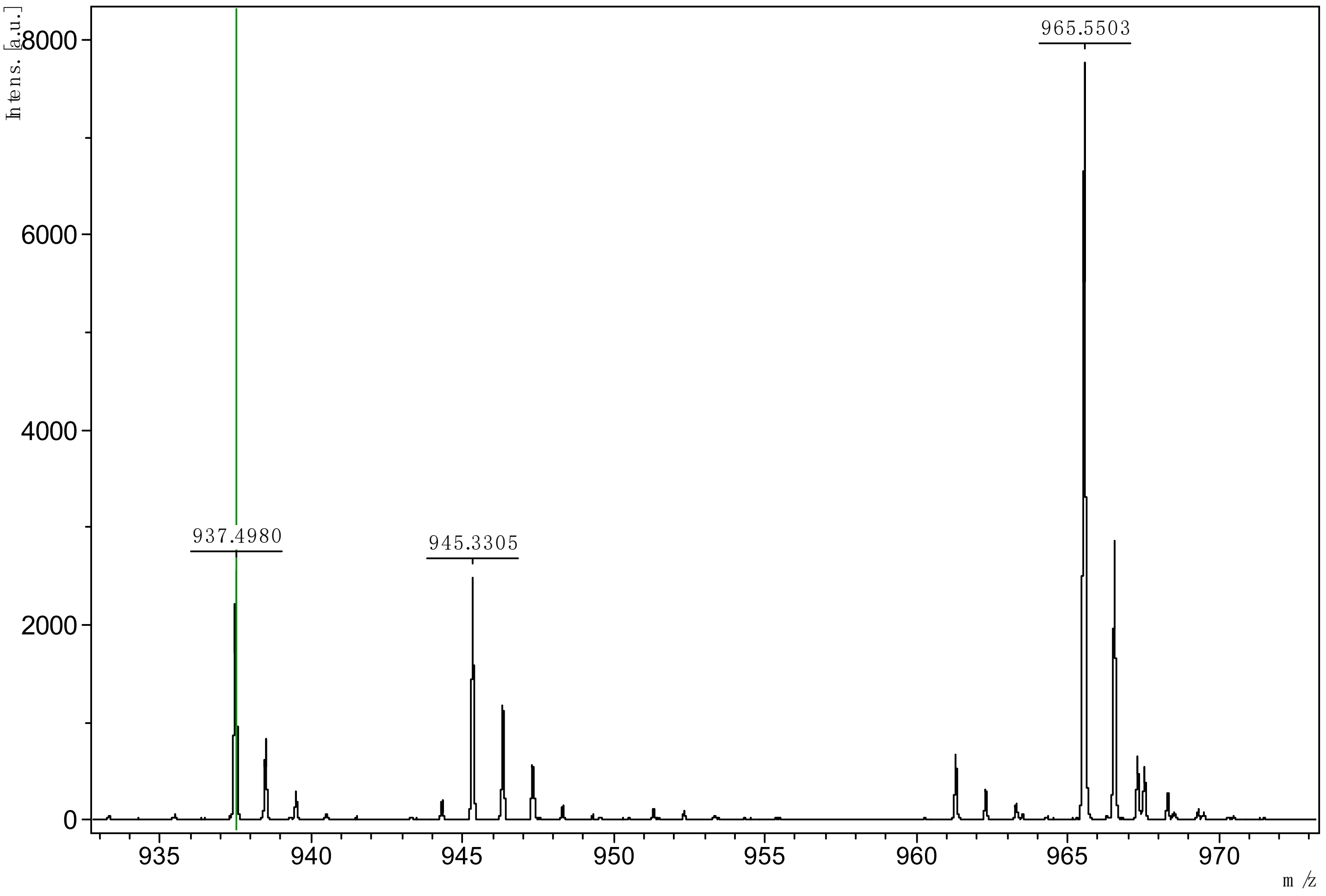

Fig. S25. HRMS spectrum of compound **8** (*p*-nitroaniline, standard).

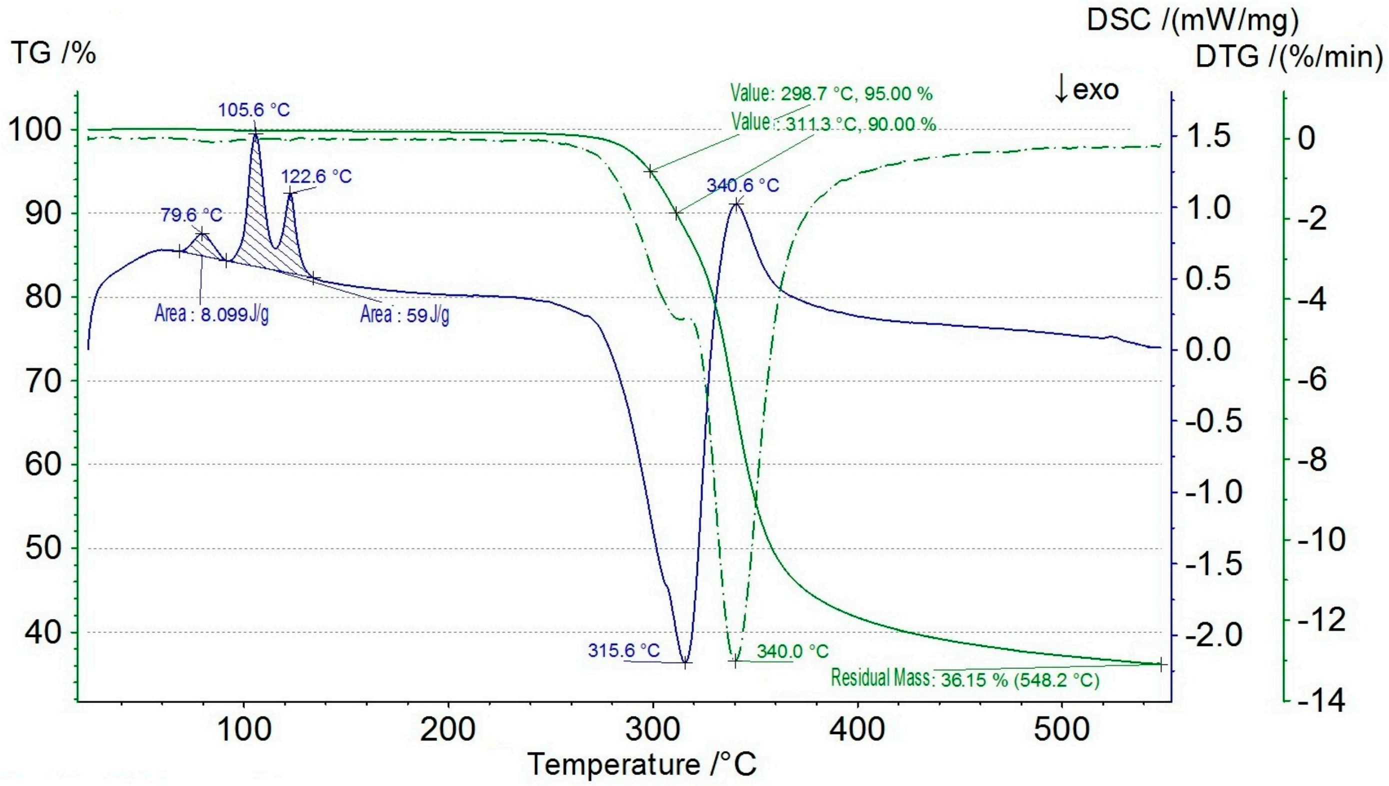

Fig. S26. TG/DTG-DSC curves of compound **8**.

Compound 8’

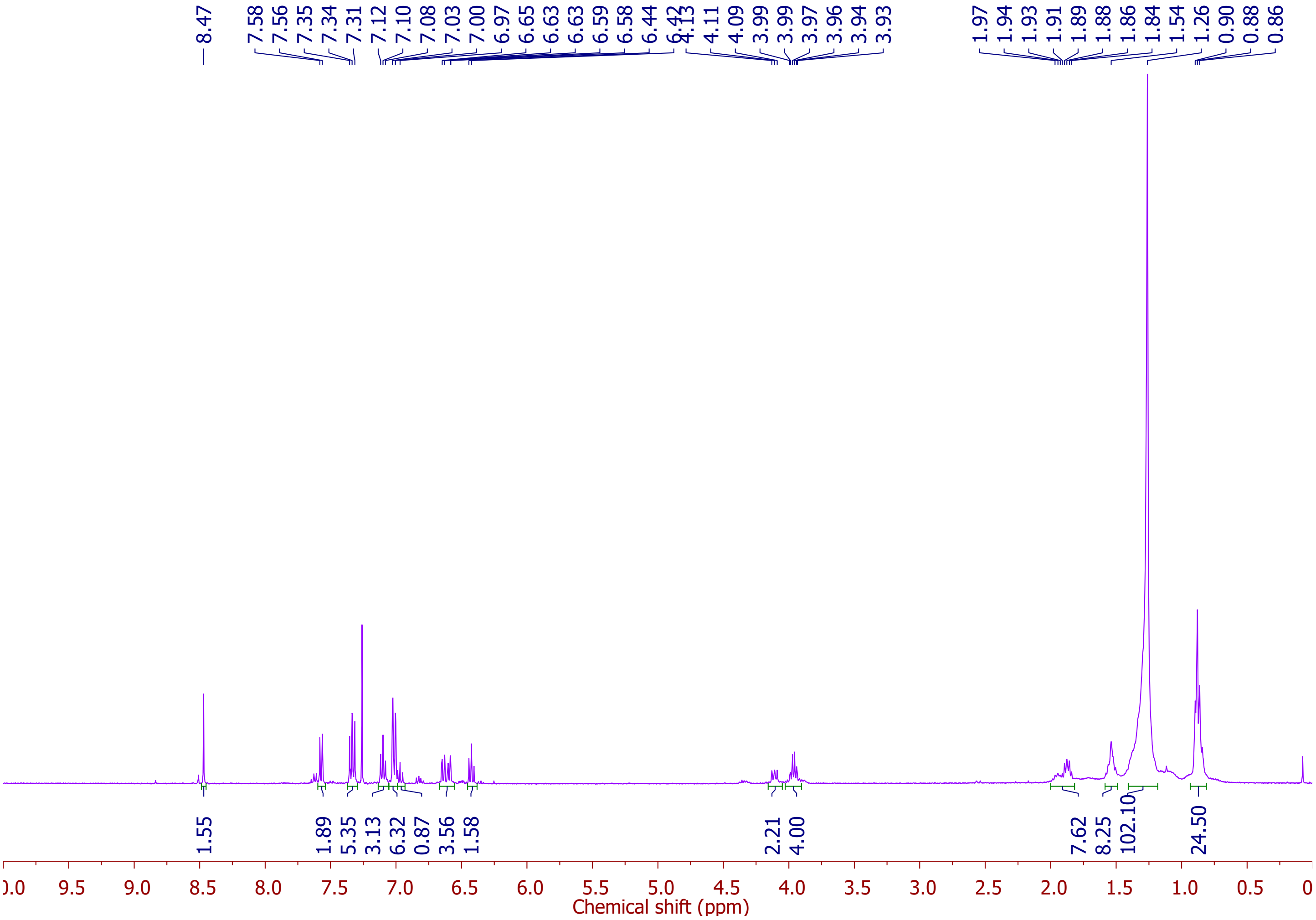

Fig. S27. ^{1}H NMR spectrum of compound **8’** ($CDCl_3$, 400 MHz, 298 K).

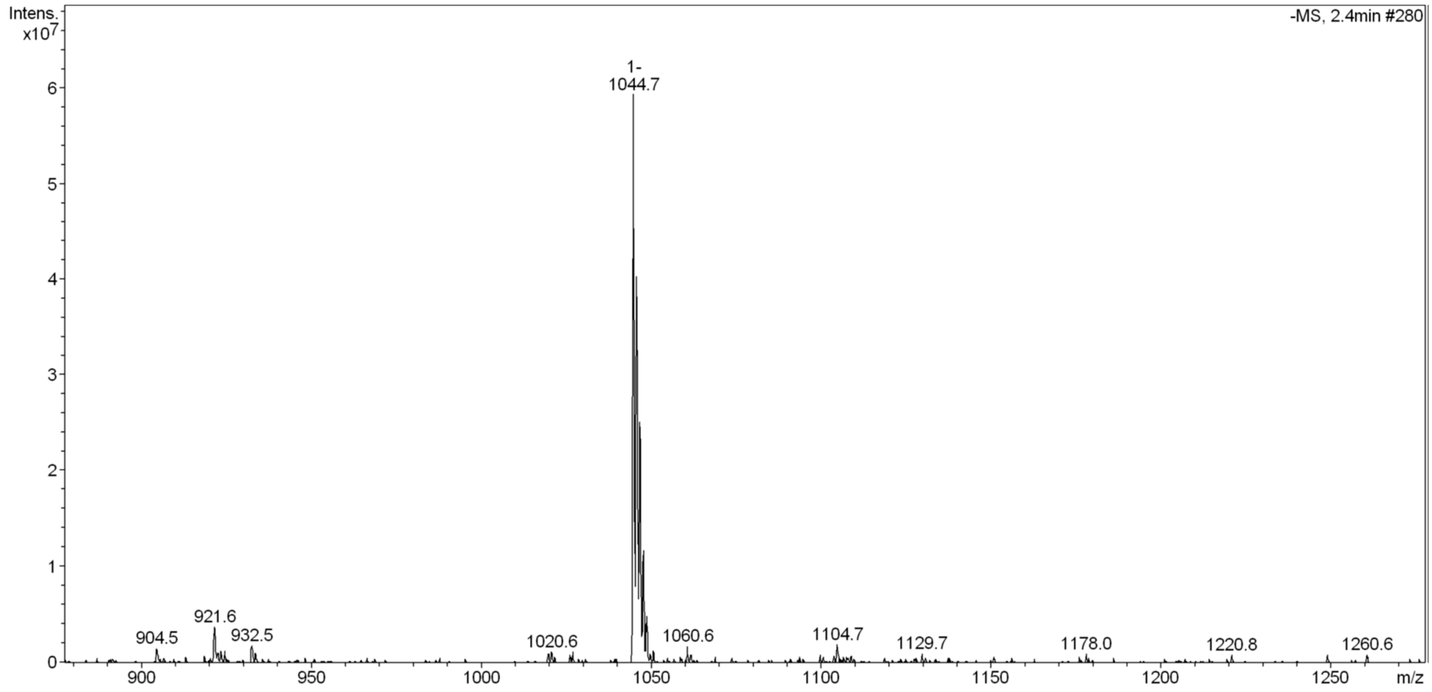

Fig. S28. ESI mass spectrum of compound **8’** in negative-ion recording mode.

Compound 10

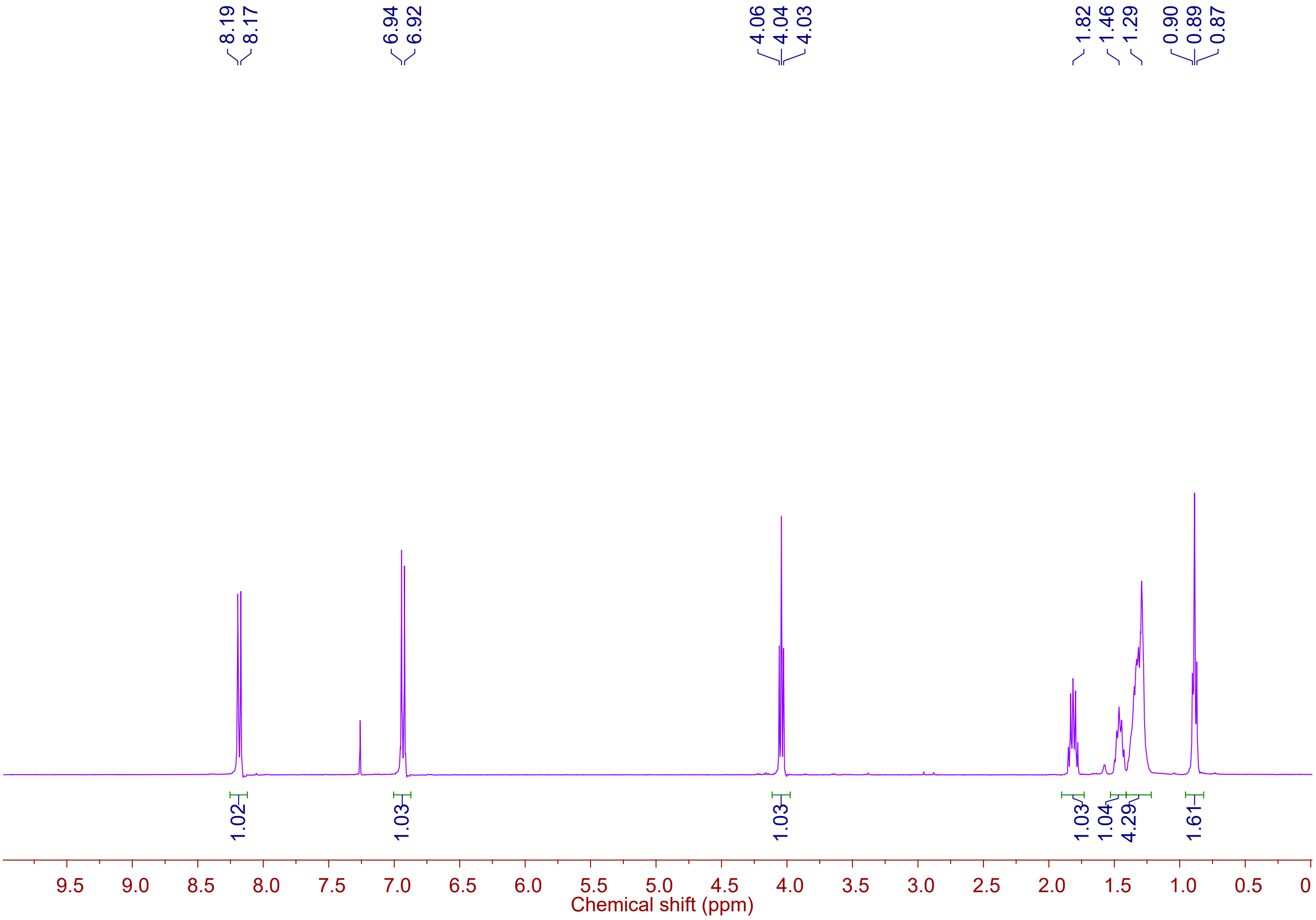

Fig. S29. ^{1}H NMR spectrum of compound **10** ($CDCl_3$, 400 MHz, 298 K).

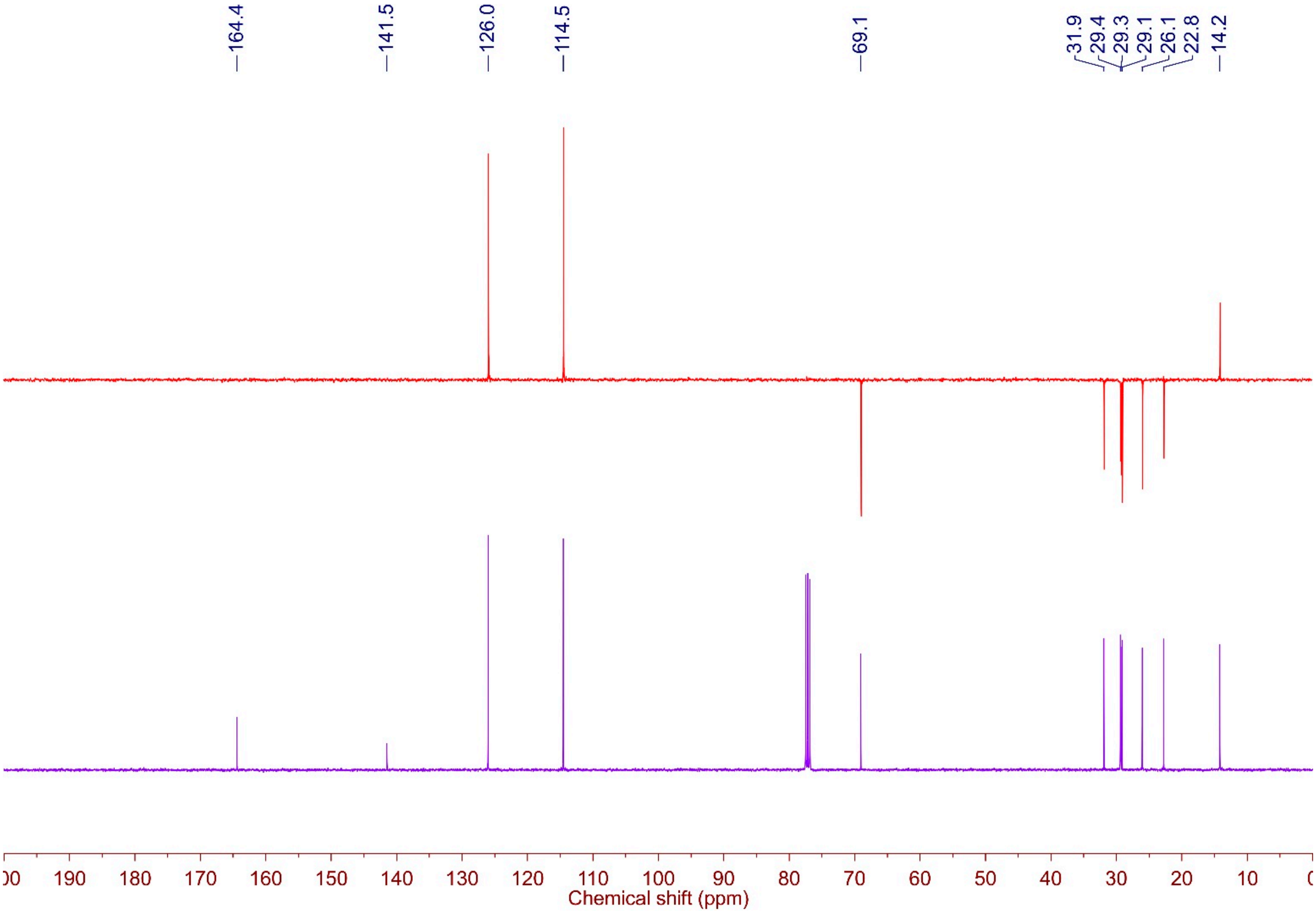

Fig. S30. ^{13}C NMR spectrum and DEPT-135 experiment of compound **10** ($CDCl_3$, 100 MHz, 298 K).

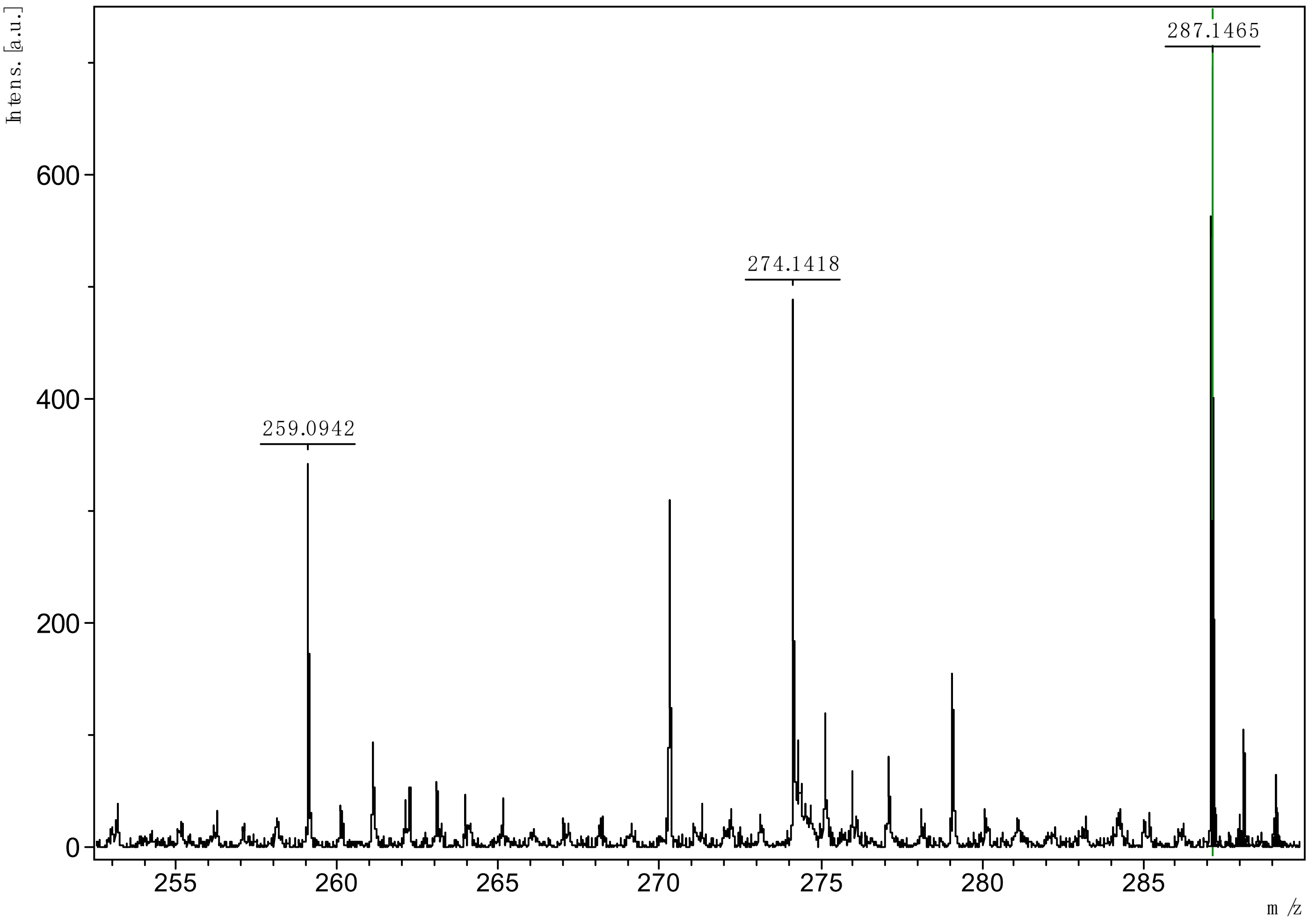

Fig. S31. HRMS spectrum of compound **10** (*p*-nitroaniline, standard).

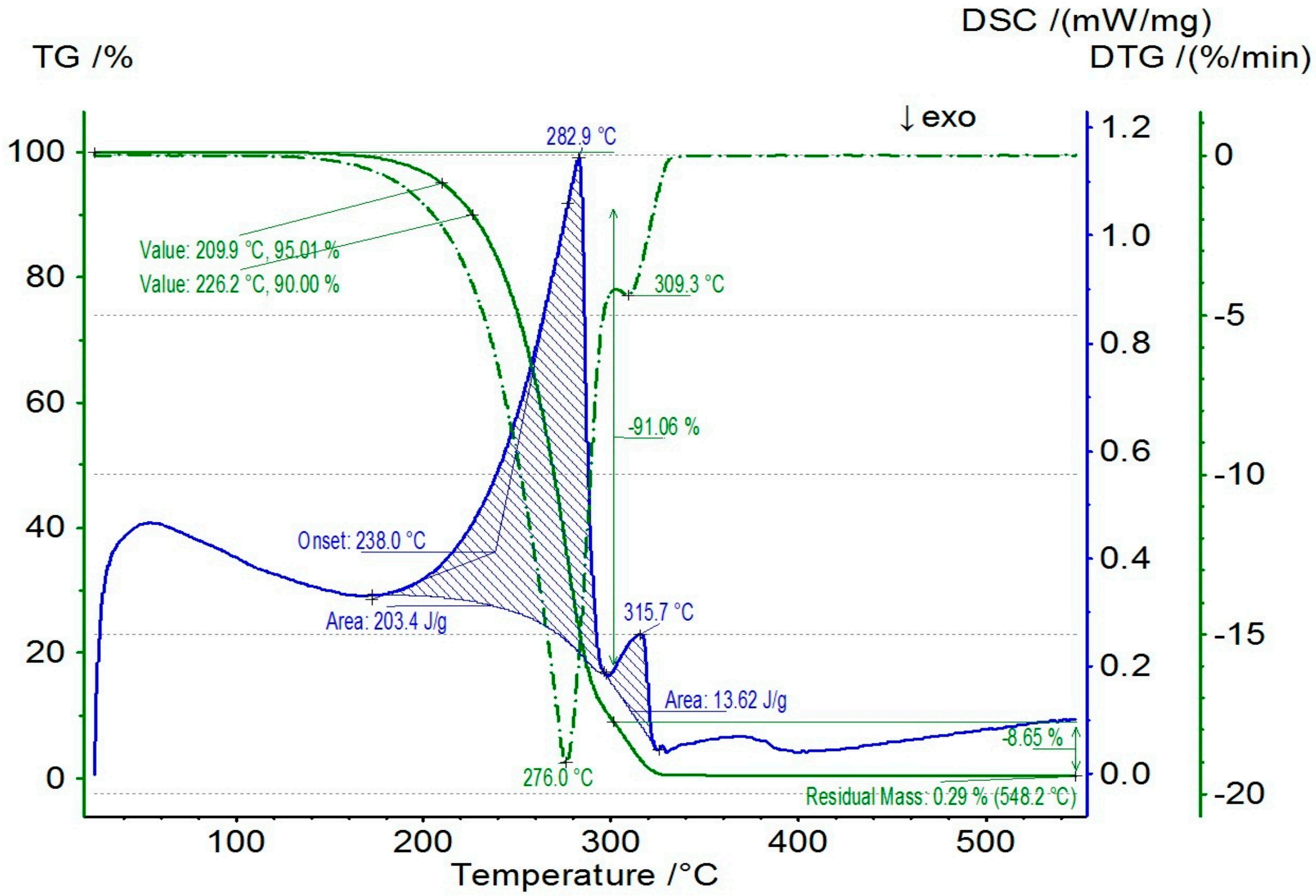

Fig. S32. TG/DTG-DSC curves of compound **10**.

Compound 12

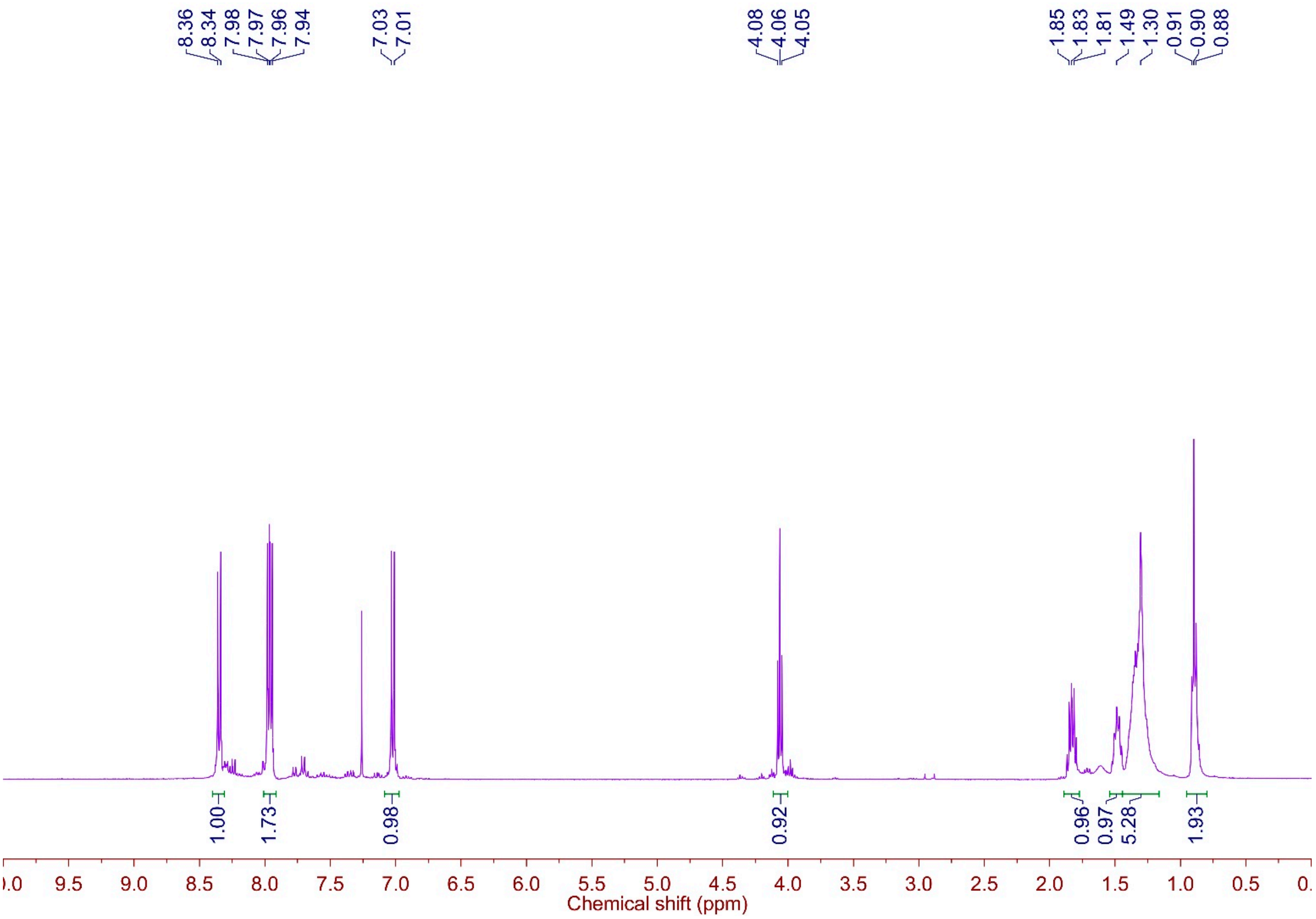

Fig. S33. ^{1}H NMR spectrum of compound **12** ($CDCl_3$, 400 MHz, 298 K).

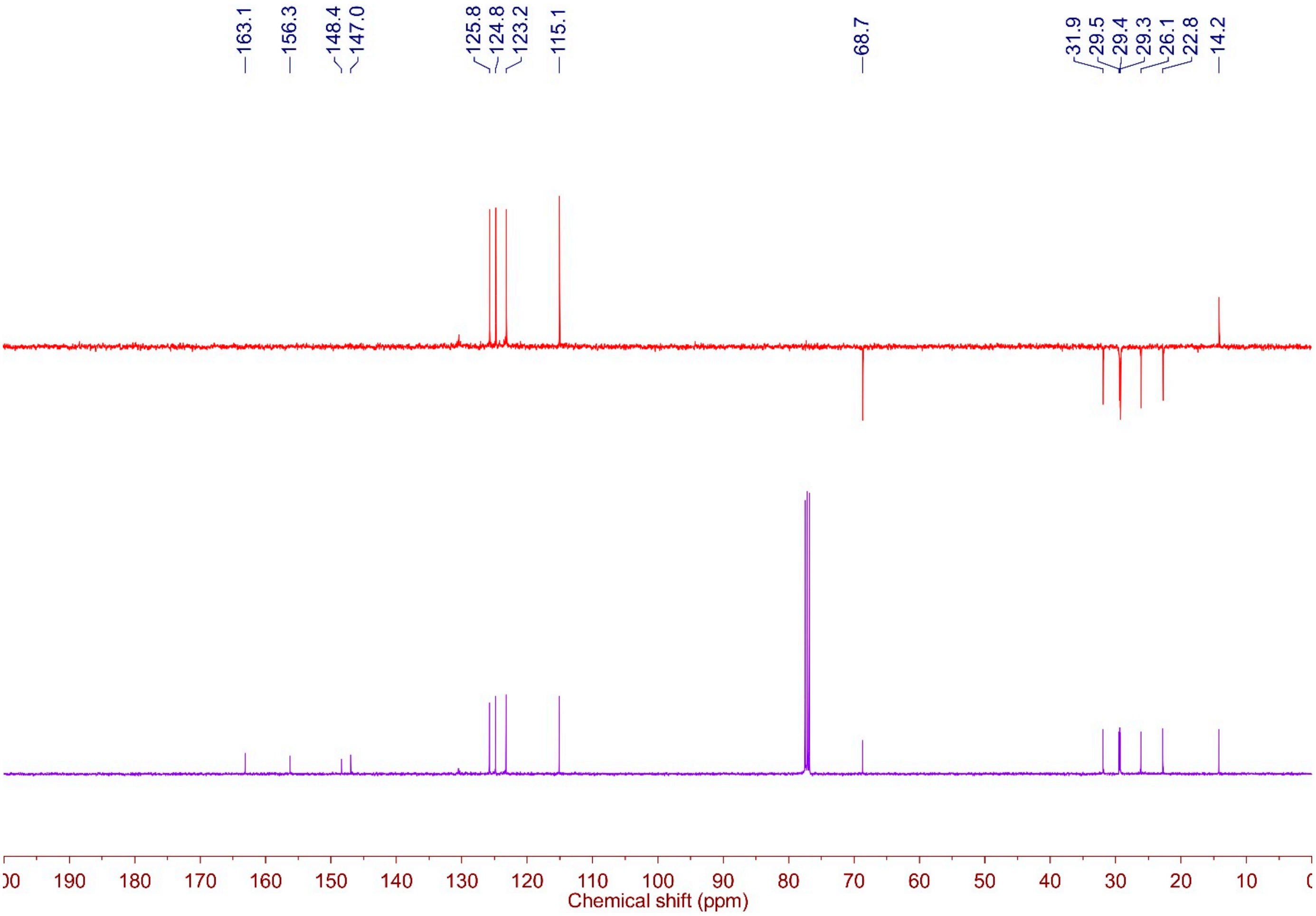

Fig. S34. ^{13}C NMR spectrum and DEPT-135 experiment of compound **12** ($CDCl_3$, 100 MHz, 298 K).

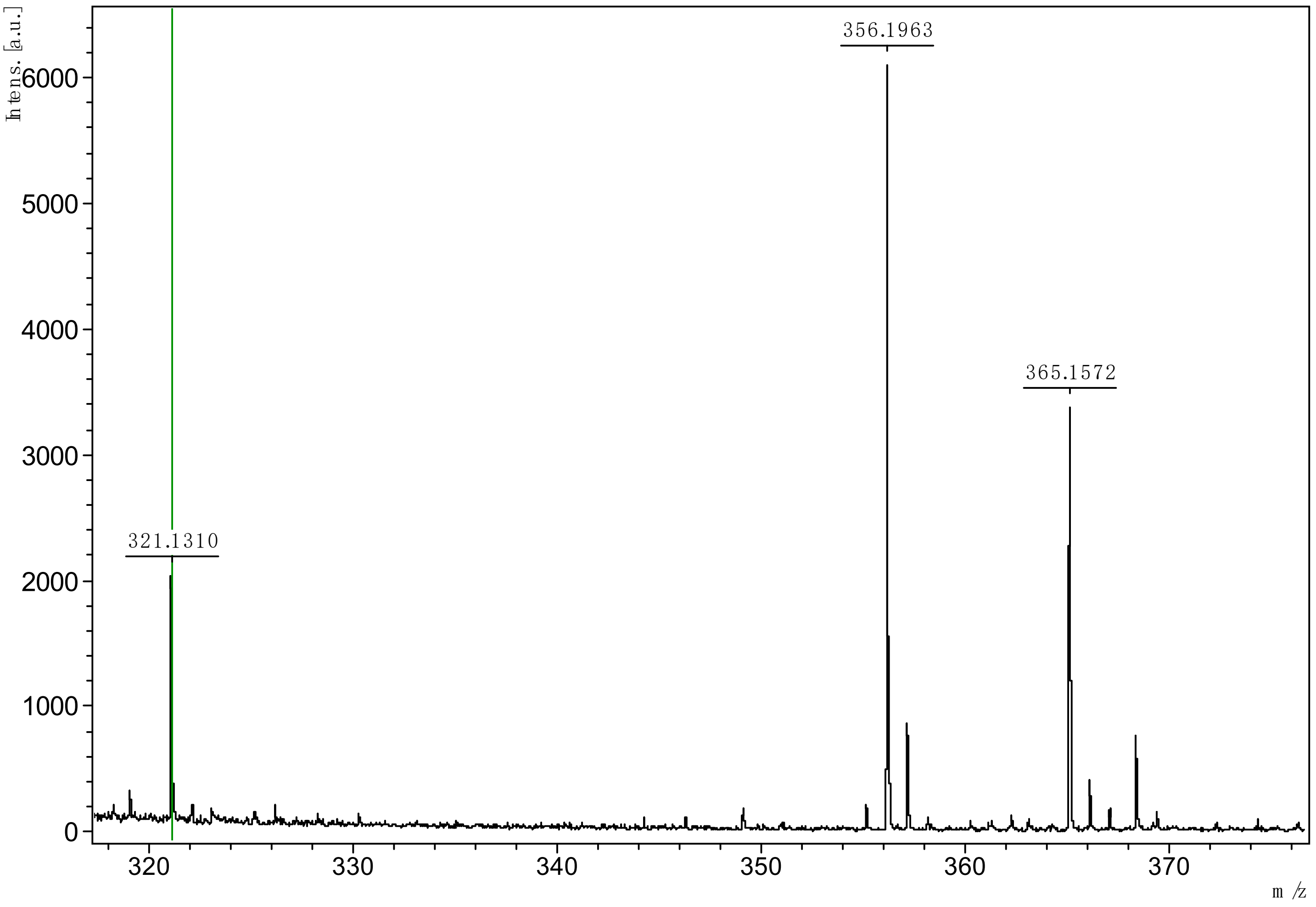

Fig. S35. HRMS spectrum of compound **12** (*p*-nitroaniline, standard).

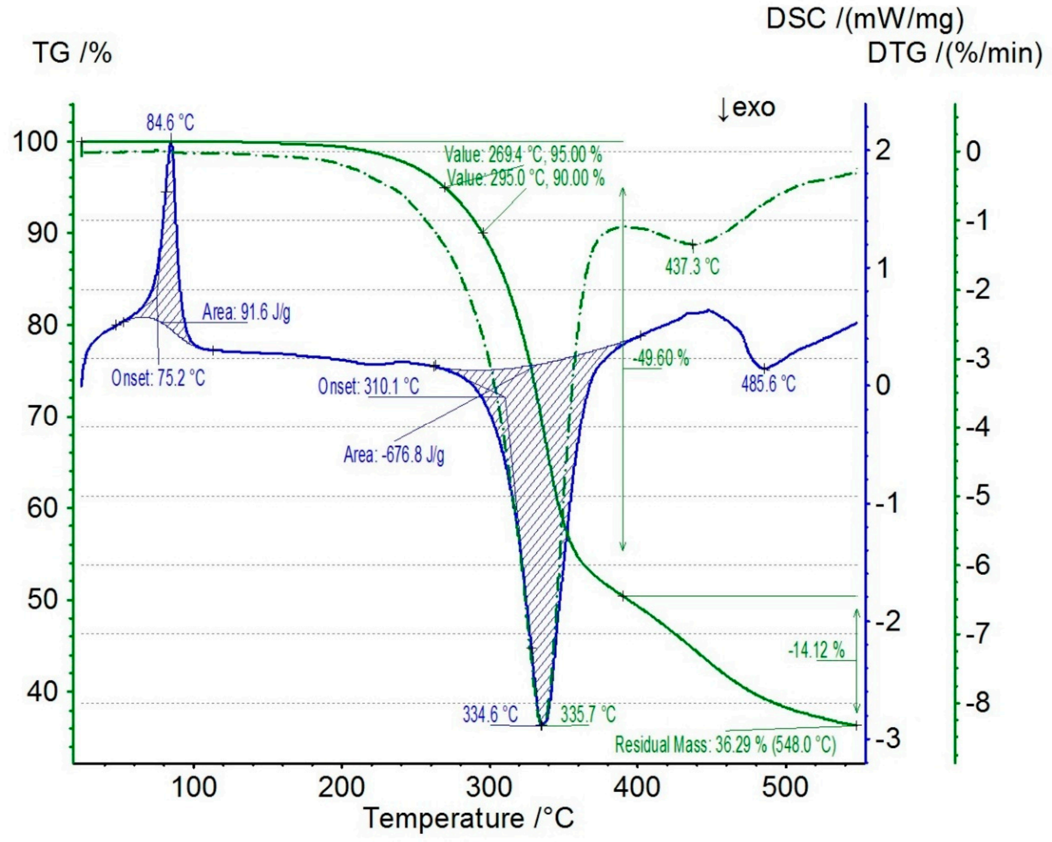

Fig. S36. TG/DTG-DSC curves of compound **12**.

Computational data of relative energies of calixarene conformers

Table S1. Energies (B3LYP/6-31+g(d)) of main conformations of compounds **2a'** and **5'** (models of compounds **2a** and **5** with C_6H_{13} as alkyl group).

Conformer	**2a'**	kcal/mol	**5'**	kcal/mol
PC	**-4107.6555171**	**0.1**	**-5469.7601573**	**0**
DC	-4107.6538975	1.1	-5469.7589047	0.8
1,3-alt	-4107.6496971	3.7	-5469.75000574	6.4
1,2-alt	**-4107.6556651**	**0**	-5469.75893876	0.8
paco	-4107.6534106	1.4	-5469.7559421	2.6

TG-DSC spectra of precursors

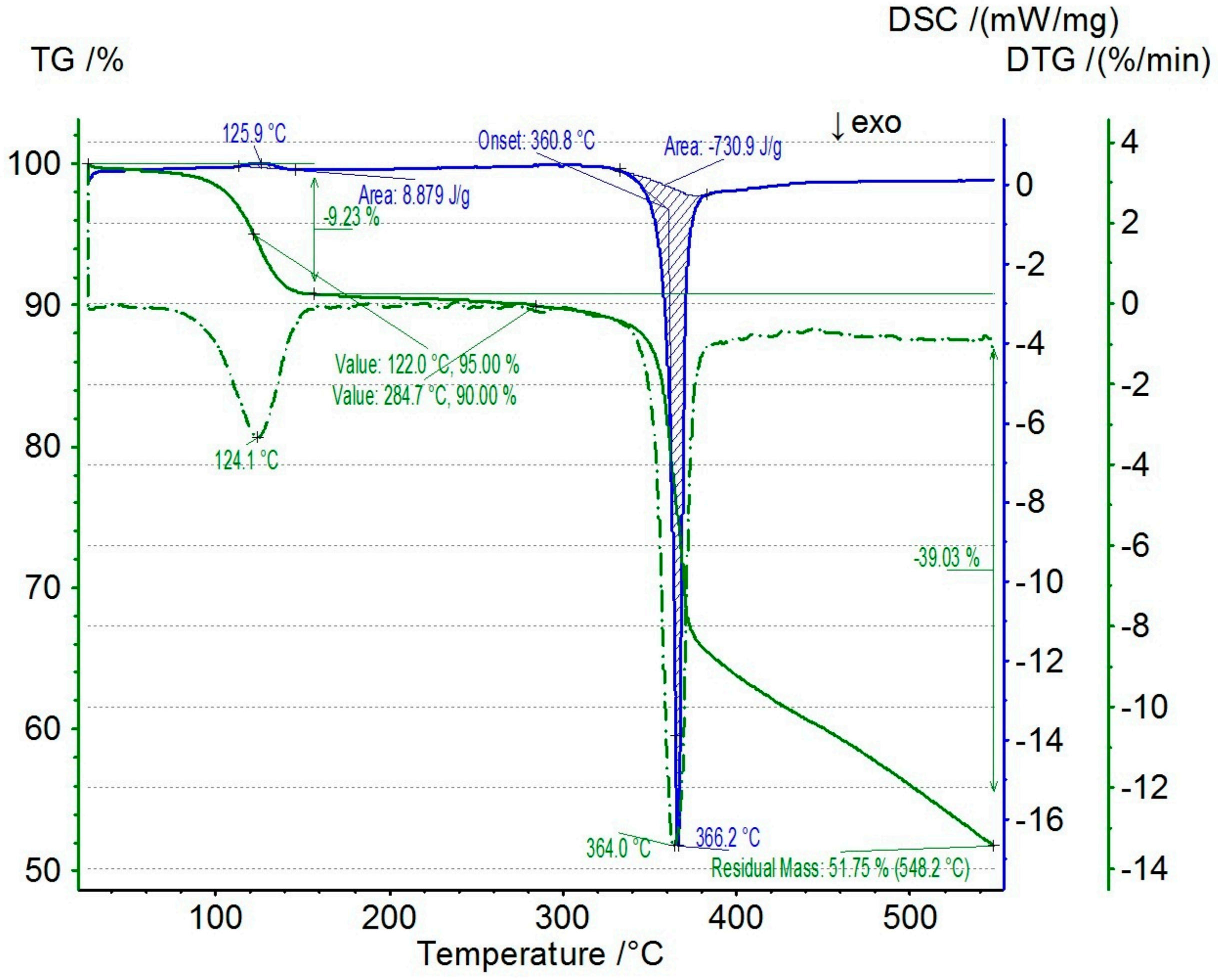

Fig. S37. TG/DTG-DSC curves of compound **1**.

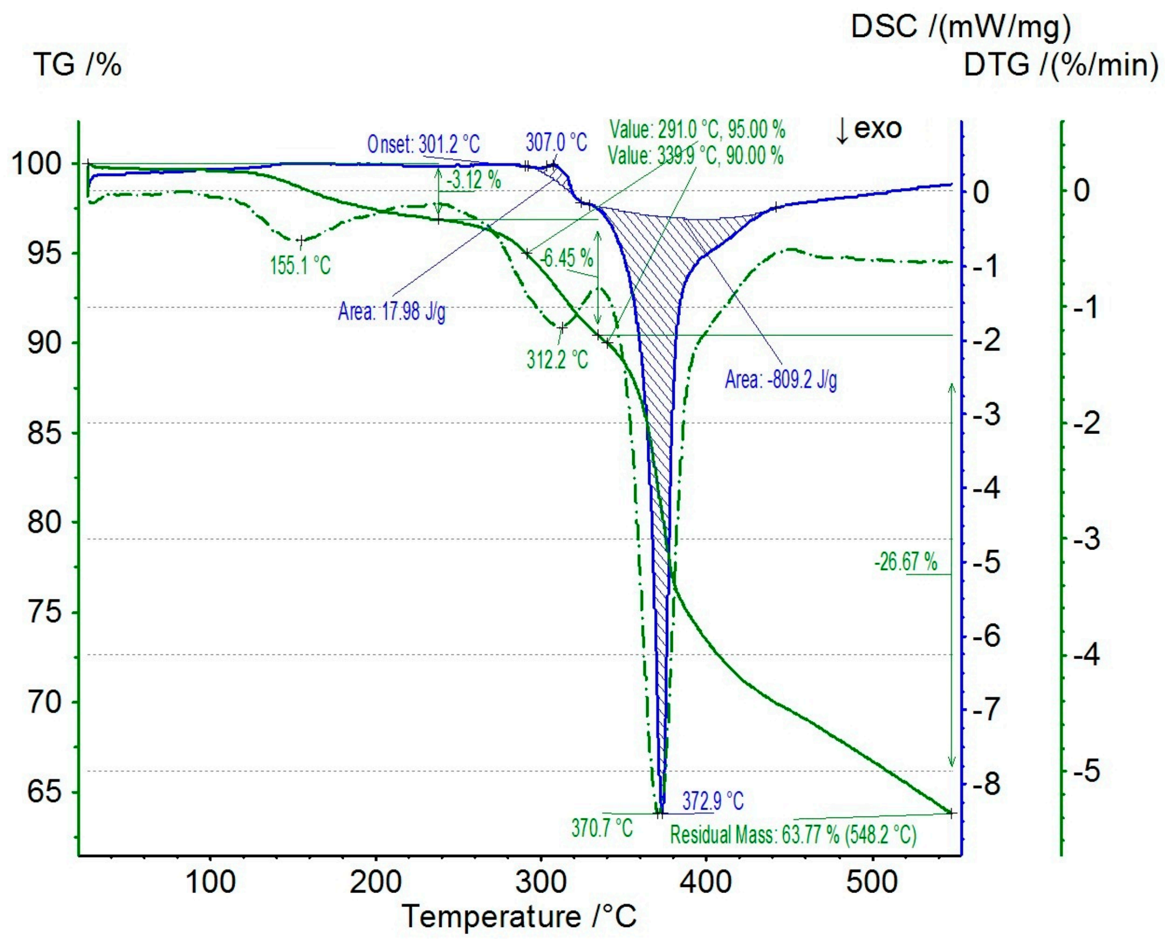

Fig. S38. TG/DTG-DSC/dDSC curves of compound **4**.

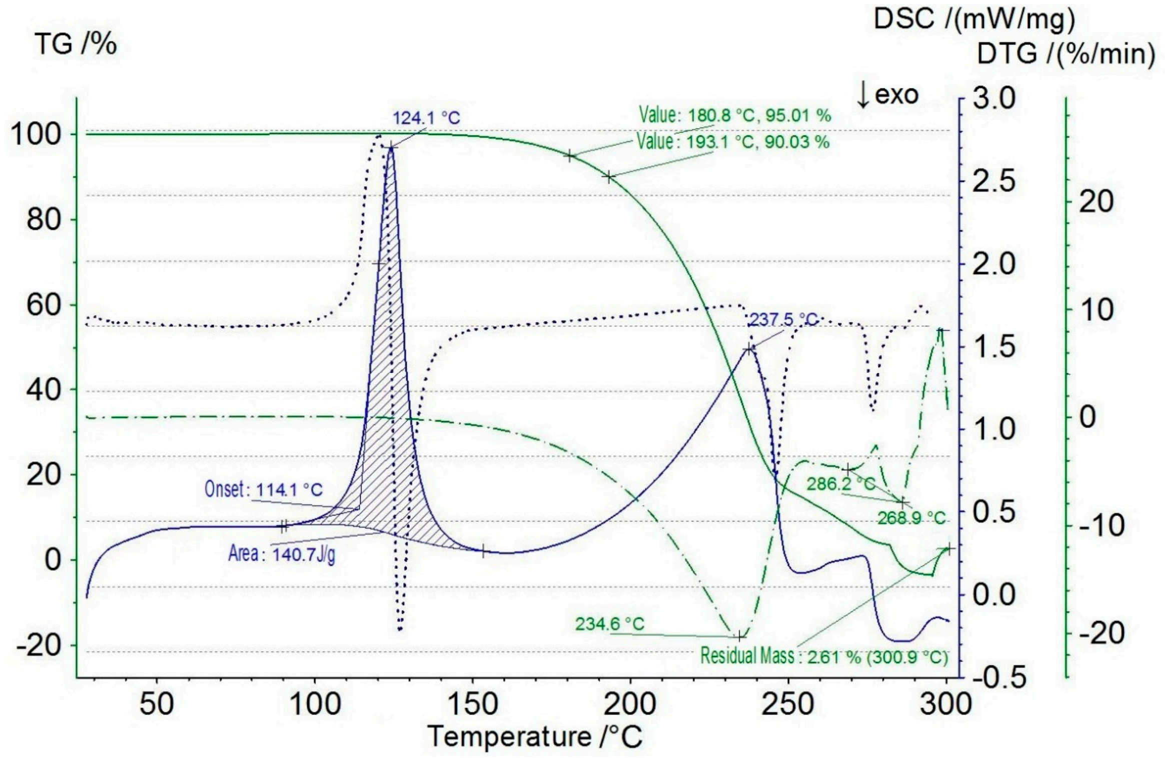

Fig. S39. TG/DTG-DSC/dDSC curves of compound **9**.

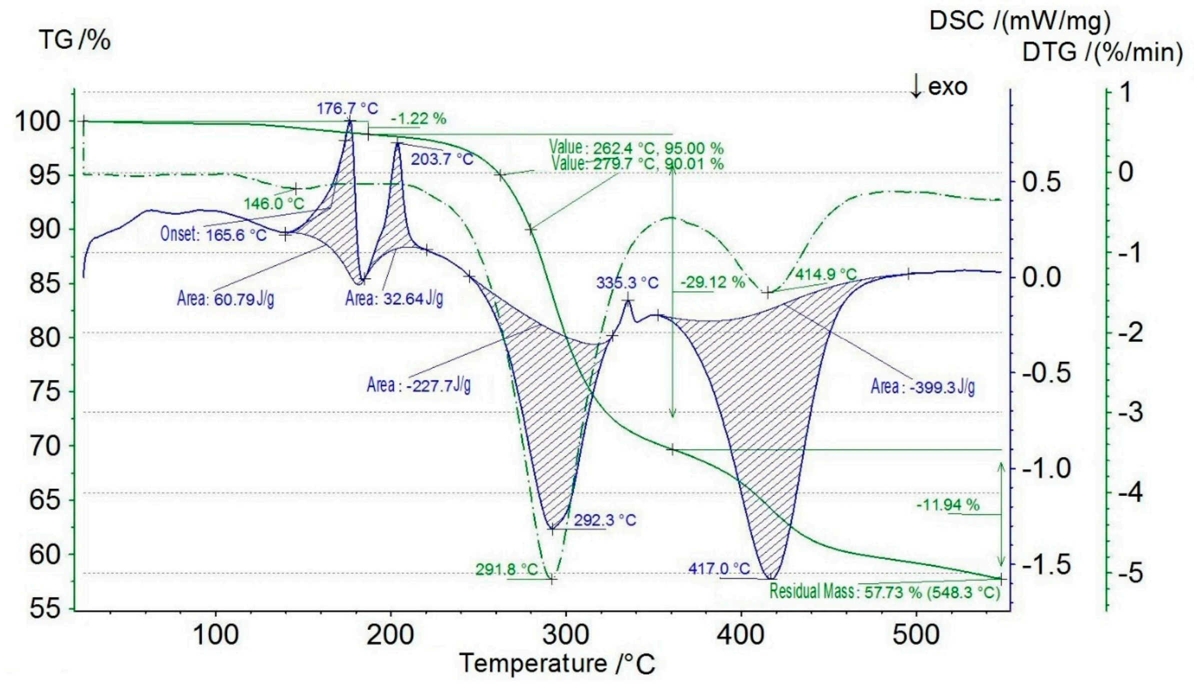

Fig. S40. TG/DTG-DSC curves of compound **11**.

UV/Vis spectra of chromophores in solution and at air–water interface

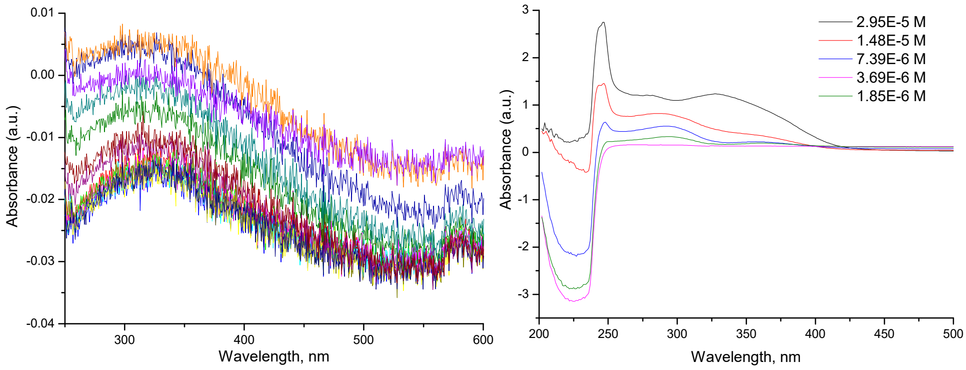

Fig. S41. (a) Reflection-absorption spectra of compound **1** at the air–water interface with the change of the surface pressure (C_1 = 0.02 mg/mL in $CHCl_3$–MeOH; V = 500 µL; t =5°C); (c) UV/Vis absorption spectra of compound **1** in $CHCl_3$–MeOH (concentrations are given in legends).

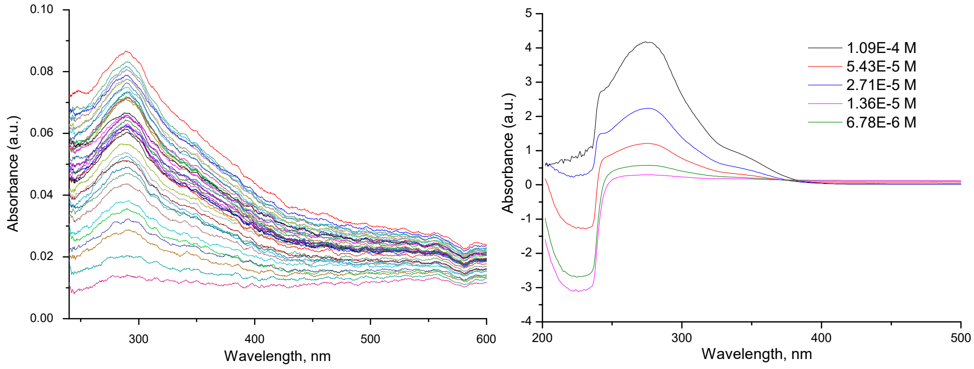

Fig. S42. (a) Reflection-absorption spectra of compound **2b** at the air–water interface with the change of the surface pressure (C_{2b} = 0.02 mg/mL in $CHCl_3$–MeOH; V = 500 µL; t =25°C); (b) UV/Vis absorption spectra of compound **2b** in $CHCl_3$–MeOH (concentrations are given in legends).

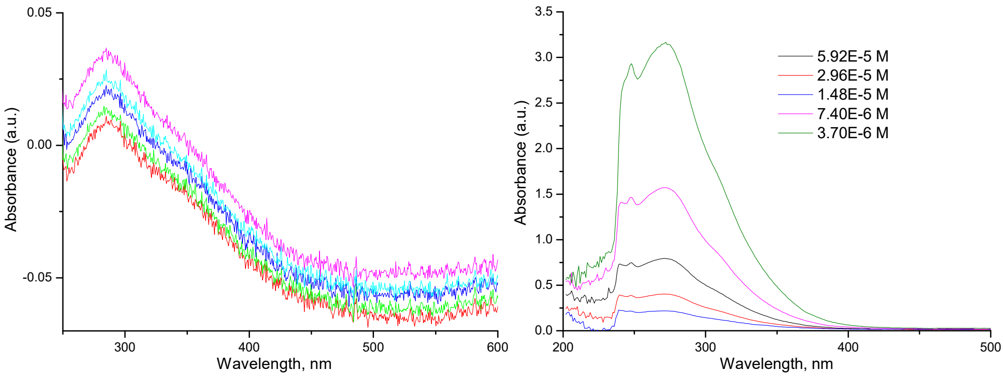

Fig. S43. (a) Reflection-absorption spectra of compound **3** at the air–water interface with the change of the surface pressure (C_3 = 0.1 mg/mL in $CHCl_3$; V = 50 µL; t =5°C); (b) UV/Vis absorption spectra of compound **3** in $CHCl_3$ (concentrations are given in legends).

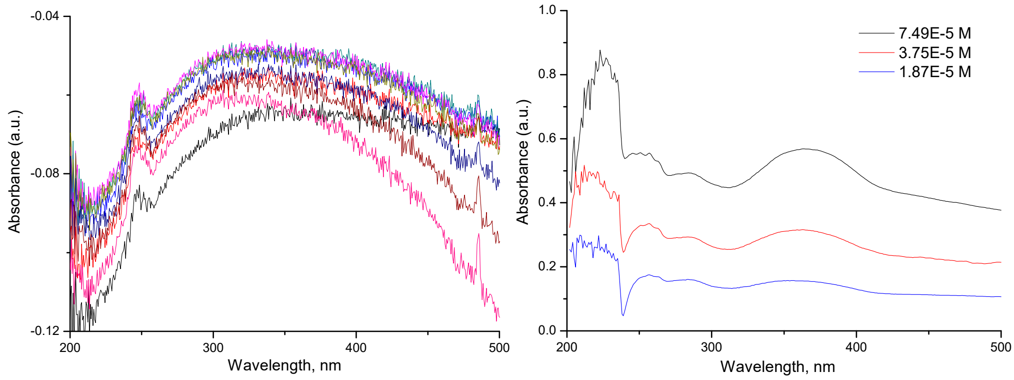

Fig. S44. (a) Reflection-absorption spectra of compound **4** at the air–water interface with the change of the surface pressure (C_4 = 0.09 mg/mL in 90%$CHCl_3$–10%MeOH; V = 150 µL; t =25°C); (b) UV/Vis absorption spectra of compound **4** in 90%$CHCl_3$–10%MeOH (concentrations are given in legends).

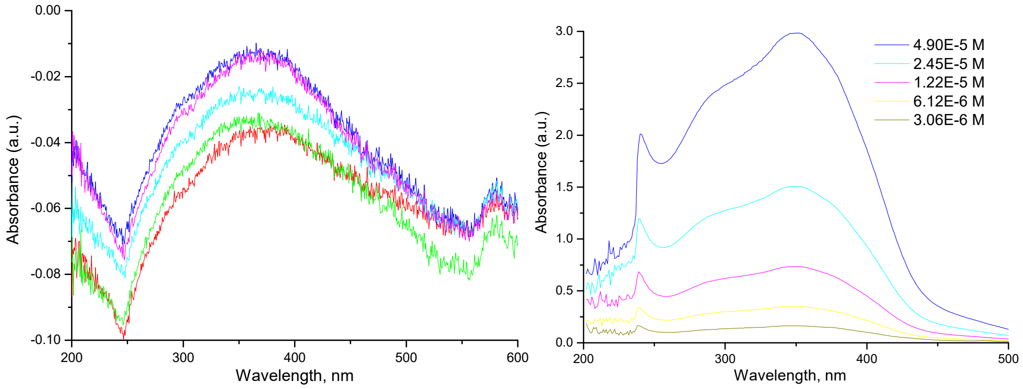

Fig. S45. (a) Reflection-absorption spectra of compound **5** at the air–water interface with the change of the surface pressure (C_5 = 0.068 mg/mL in $CHCl_3$; V = 90 μL; t =25°C); (b) UV/Vis absorption spectra of compound **5** in $CHCl_3$ (concentrations are given in legends).

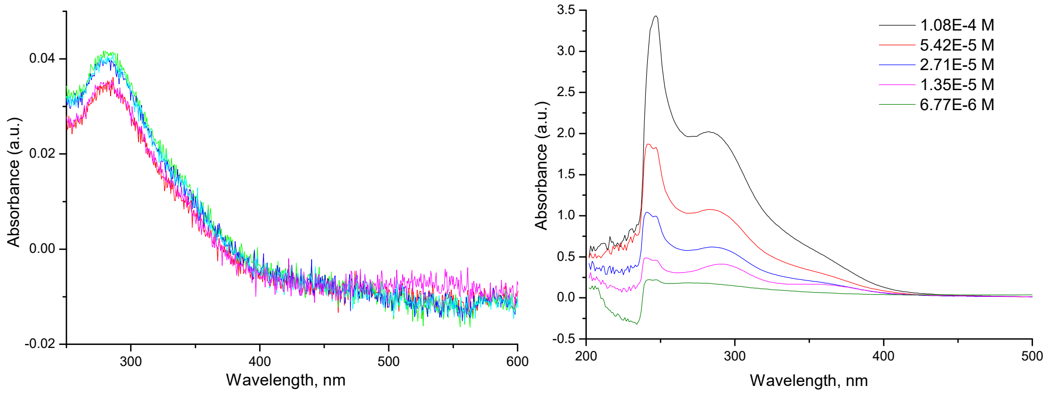

Fig. S46. (a) Reflection-absorption spectra of compound **8** at the air–water interface with the change of the surface pressure (C_8 = 0.1 mg/mL in $CHCl_3$; V = 50 μL; t =25°C); (b) UV/Vis absorption spectra of compound **8** in $CHCl_3$ (concentrations are given in legends).

Powder X-ray diffractometry data

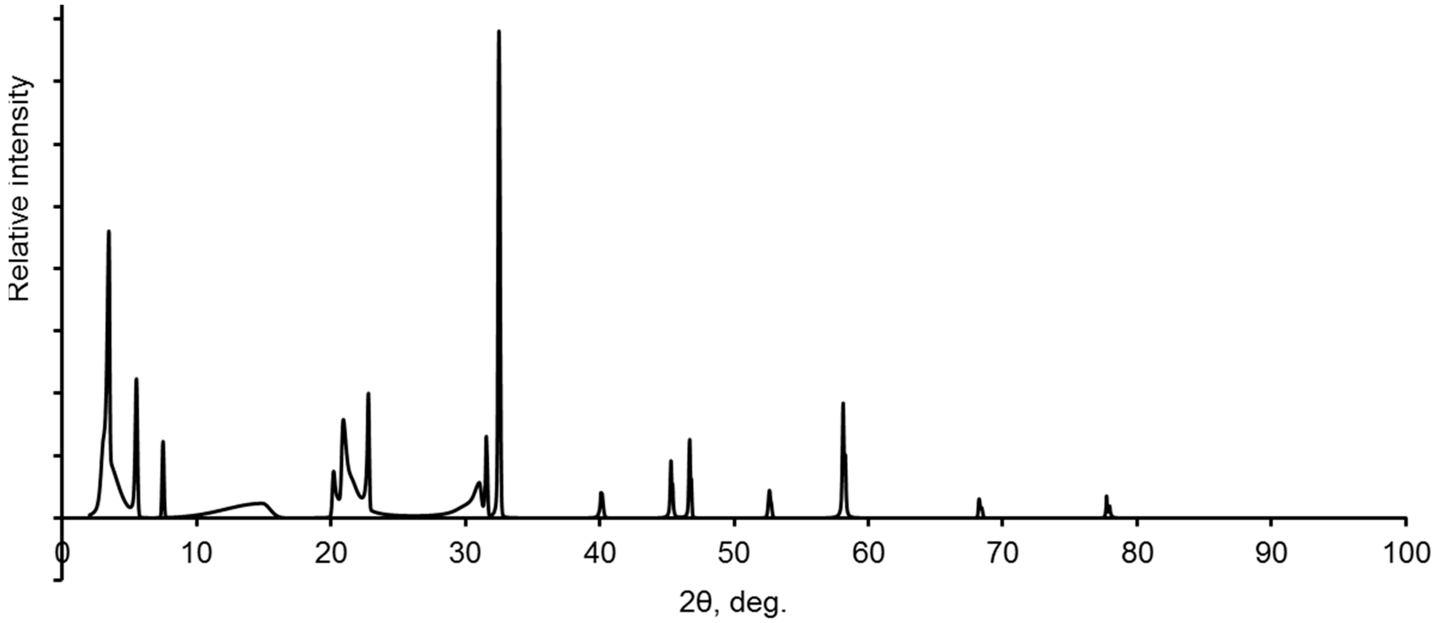

Fig. S47. Powder X-ray diffractogram of calixarene **3**.

Computational data of UV/Vis spectra of nitrothiacalixarene 3

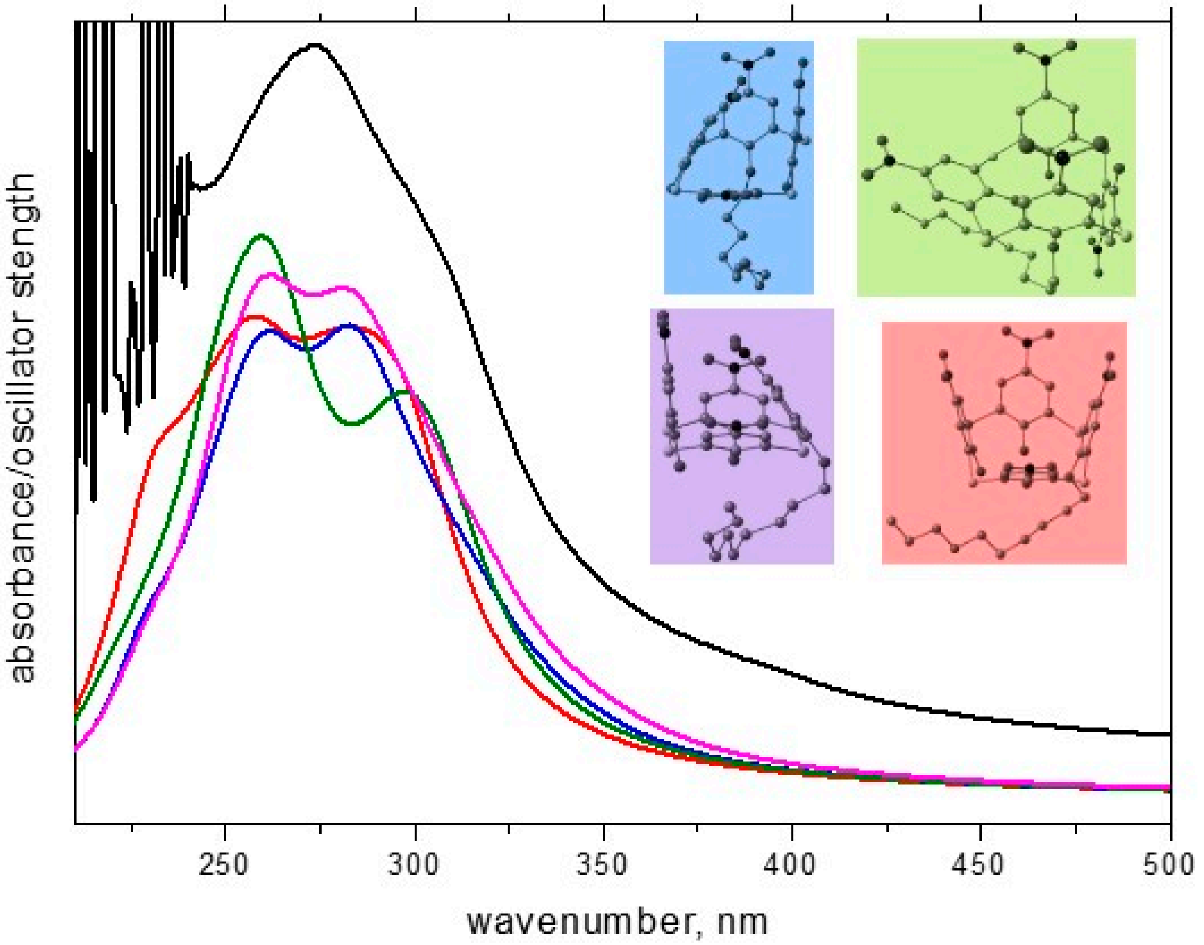

Fig. S48. Experimental (DCM solution, $C = 10^{-5}$ mol/L, black line) and calculated UV/Vis absorption spectra of calixarene **3**: *distorted cone 1* (blue), *distorted cone 2* (violet), *partial cone* (green) and *pinched cone* (red).

Table S2. Calculated excitation energies (nm), oscillator strength (f), and assignments of the longest-wavelength electronic transitions of dimers **a**–**e**.

Dimer	λ, nm	f	Assignment	Plots of frontier orbitals, mostly contributing the transition	
**a**	359	0.0045	HOMO->LUMO	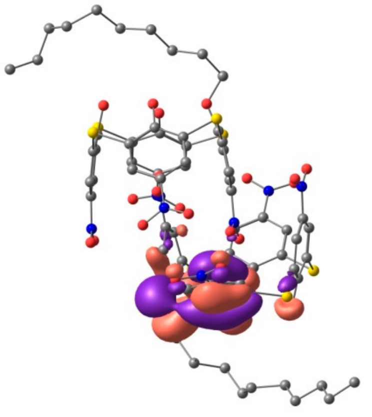	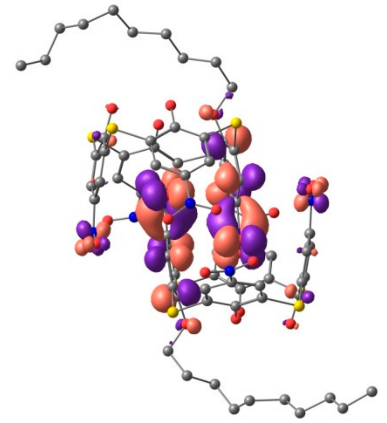
**b**	349	0.0702	HOMO->LUMO+1	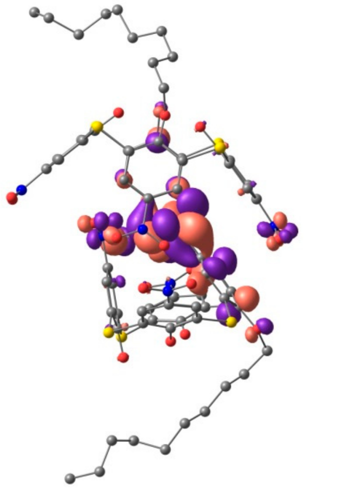	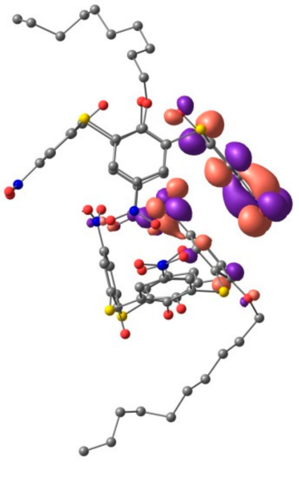

			HOMO-1->LUMO+1	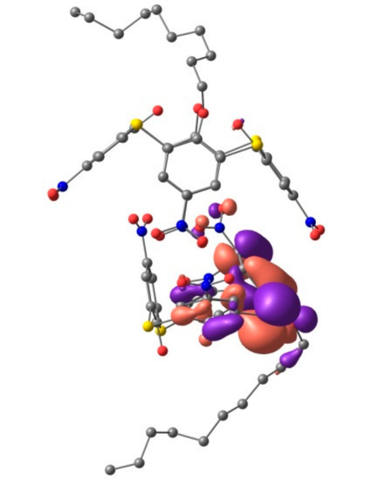	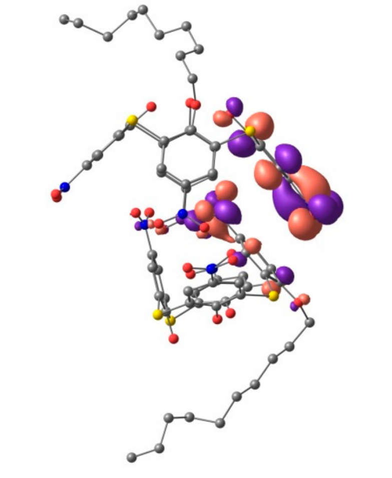
**c**	377	0.0038	HOMO-1->LUMO	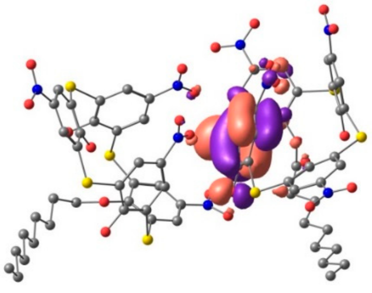	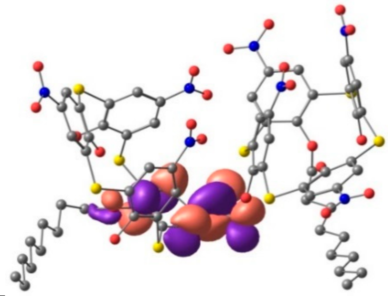
**d**	341	0.023	HOMO-1->LUMO	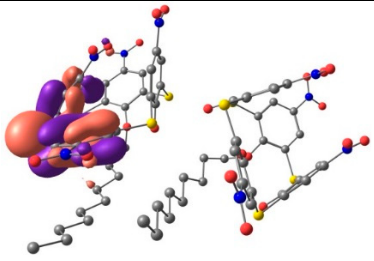	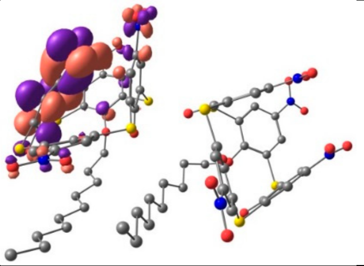
**e**	337	0.0205	HOMO->LUMO+1	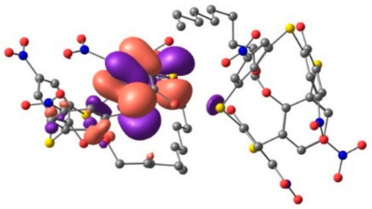	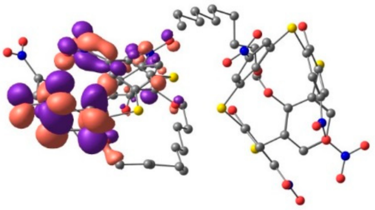
